# BAP1 regulates HSF1 activity and cancer immunity in pancreatic cancer

**DOI:** 10.1186/s13046-024-03196-4

**Published:** 2024-09-30

**Authors:** Weiwei Yuan, Qiyue Zhang, Yuhan Zhao, Wentao Xia, Shilin Yin, Xueyi Liang, Taoyu Chen, Gaofeng Li, Yanshen Liu, Zhiqiang Liu, Jinxi Huang

**Affiliations:** 1 Department of General Surgery, The Affiliated Cancer Hospital of Zhengzhou University & Henan Cancer Hospital, Zhengzhou, 450008 China; 2 Department of Pancreatic Surgery, Union Hospital, Tongji Medical College, Huazhong University of Science and Technology, Wuhan, 430022 China; 3 Sino-German Laboratory of Personalized Medicine for Pancreatic Cancer, Union Hospital, Tongji Medical College, Huazhong University of Science and Technology, Wuhan, 430022 China; 4 Cancer Center, Union Hospital, Tongji Medical College, Huazhong University of Science and Technology, Wuhan, 430022 China

**Keywords:** BRCA1 associated protein-1, Programmed death-ligand 1, Heat shock transcription factor 1, Pancreatic cancer

## Abstract

**Background:**

The vast majority of pancreatic cancers have been shown to be insensitive to single-agent immunotherapy. Exploring the mechanisms of immune resistance and implementing combination therapeutic strategies are crucial for PDAC patients to derive benefits from immunotherapy. Deletion of BAP1 occurs in approximately 27% of PDAC patients and is significantly correlated with poor prognosis, but the mechanism how BAP1-deletion compromises survival of patients with PDAC remain a puzzle.

**Methods:**

Bap1 knock-out KPC (KrasG12D/+; LSLTrp53R172H/+; Pdx-1-Cre) mice and control KPC mice, syngeneic xenograft models were applied to analysis the correlation between BAP1 and immune therapy response in PDAC. Immunoprecipitation, RT-qPCR, luciferase and transcriptome analysis were combined to revealing potential mechanisms. Syngeneic xenograft models and flow cytometry were constructed to examine the efficacy of the inhibitor of SIRT1 and its synergistic effect with anti-PD-1 therapy.

**Result:**

The deletion of BAP1 contributes to the resistance to immunotherapy in PDAC, which is attributable to BAP1’s suppression of the transcriptional activity of HSF1. Specifically, BAP1 competes with SIRT1 for binding to the K80 acetylated HSF1. The BAP1-HSF1 interaction preserves the acetylation of HSF1-K80 and promotes HSF1-HSP70 interaction, facilitating HSF1 oligomerization and detachment from the chromatin. Furthermore, we demonstrate that the targeted inhibition of SIRT1 reverses the immune insensitivity in BAP1 deficient PDAC mouse model.

**Conclusion:**

Our study elucidates an unrevealed mechanism by which BAP1 regulates immune therapy response in PDAC via HSF1 inhibition, and providing promising therapeutic strategies to address immune insensitivity in BAP1-deficient PDAC.

**Supplementary Information:**

The online version contains supplementary material available at 10.1186/s13046-024-03196-4.

## Introduction

In the era where immunotherapy and targeted therapy have made significant strides in treating various cancer types, pancreatic ductal adenocarcinoma (PDAC) remains a formidable challenge, with conventional cytotoxic agents still serving as the primary treatment modality [1]. Although, the neoadjuvant chemotherapy has significantly increased the rate of surgical resection and improved patient prognosis, the limited chemotherapy options, scarce targeted therapies, and a five-year survival rate less than 10% underscore the urgent need for alternative treatment options for PDAC [2].

While data indicated the effectiveness of immunotherapy in PDAC patients with microsatellite instability-high (MSI-H), this subgroup constitutes less than 1% of cases [3]. The vast majority of pancreatic cancers remain immune cold tumors, rendering patients unable to benefit from single-agent immunotherapy. Combining immunotherapy with standard chemotherapy or dual immune checkpoint blockade strategies are also considered to yield minimal efficacy [4, 5]. Investigating the mechanisms underlying different subtypes of PDAC and developing personalized treatment strategies might be a viable approach to improving the current status of immunotherapy in PDAC.

BRCA1 associated protein-1 (BAP1) is a member of the ubiquitin C-terminal hydrolase (UCH) domain containing deubiquitinase (DUB) family [6]. It’s recognized by its significant role in governing genome stability [7], transcriptional regulation [8], cell death and metabolism [9]. In contrast to the situation observed in mesothelioma and renal clear cell carcinoma (ccRCC), where BAP1 function is commonly affected by mutations [10], in pancreatic cancer, approximately 26% of patients exhibit low expression of BAP1 due to copy number deletions [11].

BAP1’s involvement in cancer immunity was initially underreported. Observations in ccRCC revealed that loss-of-function mutations of BAP1 upregulated the immunosuppressive gene CCR5, resulting in resistance to immune checkpoint blockade (ICB) therapy [12]. And the inactivation of BAP1 in pancreatic progenitor cells regulated the infiltration of immune cells [11]. However, the mechanisms by which BAP1 loss or mutations regulate cancer immunity remain unclear.

Heat shock transcription factor 1 (HSF1) is widely recognized as pivotal for cellular responses to diverse stresses, preserving cell function and viability through the induction of genes associated with protein quality control [13, 14], which also solidifies its role as a significant facilitator of various cancers and a critical determinant of therapeutic efficacy, including pancreatic cancer [15, 16]. It is noteworthy that HSF1 has recently been discovered to play a significant regulatory role in tumor immunity, and is recognized as potential target in cancer immunotherapy [17, 18]. However, further exploration is needed to identify the potential beneficiaries among patient populations.

In this study, we show that BAP1 competes with SIRT1 for binding with the trimerized HSF1. The BAP1-HSF1 interaction safeguards the acetylation of HSF1 at the K80 site, promotes the monomerization of HSF1, and facilitates its dissociation from DNA. Our findings indicate that targeted inhibition of SIRT1 or HSF1 could serve as a promising strategy to sensitize BAP1-deficient pancreatic cancer subtypes to immunotherapy.

## Methods and materials

### Cell culture and transfection

The cell lines employed in this investigation, specifically 293T, CFPAC, PaTu8988, SW1990, and BxPC-3, were cultured in Dulbecco’s Modified Eagle Medium (DMEM) (Gibco, USA). This culture medium was supplemented with 10% fetal bovine serum (FBS) to facilitate optimal growth conditions. Cultures were maintained at 37 °C in a controlled humidified atmosphere containing 5% CO2, thereby providing a stable environment conducive to cell proliferation. Stringent certification protocols were adhered to in order to verify the purity of all cell lines and to ascertain their freedom from contamination.

For transfection, a precise volume of Lipofectamine 8000, specifically 1–2 µL, was employed to deliver 1 µg of plasmid into the target cells. Both Lipofectamine 8000 and the plasmid were individually diluted in Opti-MEM, a serum-free medium optimized for transfection. Subsequently, the two solutions were gently combined and allowed to incubate at room temperature for 15 min, promoting the formation of a stable DNA-lipid complex. Following the incubation period, this complex was introduced to the cells, facilitating the efficient transfer of the plasmid into the cellular cytoplasm.

### RNAi

The lentiviral packaging vectors, psPAX2 and pMD2.G, were co-transfected into 293T cells alongside the specific shRNA. To refine transfection conditions, the culture medium was refreshed with fresh DMEM supplemented with 10% FBS 24 h post-transfection. After 72 h, the medium containing the virus was harvested and employed for infecting cancer cells in the presence of polybrene (12 µg/mL) to augment viral infection efficiency. For ensuring stable integration and expression of the shRNA, a puromycin selection regimen was initiated at concentrations ranging from 3 to 5 µg/mL, commencing 48 h post-infection. The lentivirus-based small hairpin RNAs (shRNA) were generously provided by Zhou (Mayo Clinic), and the sequence information of the shRNA is elaborated in the Supplementary Table S2.

### Coimmunoprecipitation and Western blot analysis

In summary, the immunoprecipitation (IP) procedure was executed following established protocols. Cells were treated with 1 ml of IP lysis buffer (Beyotime, China) per 10^7 cells on ice for 30 min, supplemented with 1 mmol/L phenyl methyl sulfonyl fluoride (PMSF; Beyotime, China) and phosphatase inhibitor (Beyotime, China) to inhibit protein degradation and dephosphorylation. Subsequently, the cell lysate underwent centrifugation at 14,000 g at 4 °C for 10 min to pellet cellular debris and isolate soluble proteins. The resulting supernatants were incubated overnight at 4 °C with the specific primary antibody and protein A/G agarose beads (Beyotime, China) to capture the target protein-antibody complex. Subsequent washing steps were performed five times with ice-cold IP lysis buffer to eliminate unbound proteins and impurities. For Western blotting, the protein concentration in the lysates was determined using a bicinchoninic acid protein assay (Beyotime, China). Equal amounts of denatured proteins were subjected to SDS-PAGE and transferred onto a nitrocellulose filter membrane. The membrane was then blocked with 1xTBST (Servicebio, China for TBS and Biosharp, China for Tween 20) containing 5% BSA powder at room temperature for 1 h to minimize nonspecific binding. Specific primary antibodies were subsequently applied to the membrane and allowed to incubate overnight at 4 °C to facilitate antigen-antibody binding. Following this, the membrane underwent three washes with 1xTBST to remove unbound antibodies. Subsequently, specific secondary antibodies were added and incubated with the membrane at room temperature for 1 h to amplify the signal. Finally, the samples were detected using a super-sensitive electrochemiluminescence reagent, which generates a luminescent signal proportional to the amount of bound antibodies, thereby enabling quantitative analysis of protein expression. The antibodies are provided in detail in the Supplementary Table S3.

### Nuclear and cytoplasmic protein extraction

The Nuclear and Cytoplasmic Protein Extraction Kit (Beyotime, China) was utilized following the manufacturer’s detailed instructions to efficiently separate nuclear and cytoplasmic proteins from cells. This kit provides a convenient and reliable method for isolating proteins from these two distinct cellular compartments, enabling subsequent biochemical analysis. The obtained product was used to western blot or Co-IP analysis.

### In vivo tumor models

Six-week-old C57BL/6 mice was procured from Vital River Laboratories in Beijing, China, KPC (LSL-KrasG12D/+; LSL-Trp53R172H/+; Pdx-1-Cre, 8 weeks old, sex-matched) transgenic mice and Bap1 knock-out KPC mice were purchased from MODEL ORGANISMS Inc. (Shanghai, China) and housed under pathogen-free conditions. were utilized in this investigation. Ethical approval for the study was obtained from the Ethics Committee of Tongji Medical College, Huazhong University of Science and Technology. Subcutaneously, KPC/BAP1 knockout KPC cells, totaling 5 × 10^6 in 100 µL of 1 × PBS, were injected into the right flank of the mice. The allograft volume was calculated using the formula (L × W^2 × 0.5). Upon reaching a volume of 50 mm³, mice harboring similar tumor burdens were randomly assigned, utilizing simple randomization, to different experimental groups. These groups were administered either anti-PD-1 (BioXcell, Clone RMP1-14) or IgG (BioXcell, Clone 2A3), with or without concurrent treatment with EX-527 (MedChemExpress, USA). On day 24, mice were euthanized, with exceptions made for those meeting euthanasia criteria prior to the endpoint due to unforeseen events such as ulceration or infection. Subsequently, tumors were excised for flow cytometry analysis. KPC mice with/without Bap1 knock-out were randomly assigned to different experimental groups and received anti-PD-1 (BioXcell, Clone RMP1-14) or IgG (BioXcell, Clone 2A3) respectively. All experimental procedures involving mice strictly adhered to the guidelines set forth by the local ethics committee of Tongji Medical College, Huazhong University of Science and Technology, China.

### Quantitative RT-PCR

RNA extraction from cancer cells was performed using TRIzol (Thermo Fisher Scientific). Subsequently, 2 micrograms of RNA were utilized for cDNA synthesis following the guidelines outlined in the PrimeScript™ RT reagent Kit. Quantitative PCR was then conducted using the TB Green™ Fast qPCR Mix PCR kit. The fold change was determined using the ‘2-ΔΔC(T)’ method, with normalization against GAPDH. For quantification analysis, primers demonstrating an amplification efficiency within the range of 90–110% were carefully selected. The primer sequences employed for RT-qPCR are provided in detail in the Supplementary Table S2.

### RNA sequencing

A comprehensive approach was undertaken for RNA sequencing (RNA-seq), wherein 1 µg of purified RNA was dedicated per sample. The sequencing libraries were generated using the NEBNext Ultra RNA Library Prep Kit for Illumina (NEB, USA), following the manufacturer’s prescribed protocols. The clustering of the samples was performed using the cBot Cluster Generation System with the TruSeq PE Cluster Kit v3-cBot-HS (Illumina), following the manufacturer’s prescribed protocols. After clustering, the libraries were sequenced on an Illumina NovaSeq platform, generating 150-bp paired-end reads. FeatureCounts v1.5.0-p3 software was used for counting the read numbers mapped to each gene. Differential expression analysis (two biological replicates per condition) was performed using the DESeq2 (http://bioconductor.org/packages/release/bioc/html/DESeq2.html). ClusterProfiler R package was used to test the statistical enrichment of differentially expressed genes (DEGs) in Kyoto Encyclopedia of Genes and Genomes (KEGG) pathways.

### Chromatin immunoprecipitation (ChIP) and ChIP-qPCR

The Chromatin Extraction Kit (Abcam, USA) and ChIP Kit Magnetic - One Step (Abcam, USA) were employed to conduct the chromatin immunoprecipitation (ChIP) procedure. The purified DNA obtained from this process underwent analysis using methods similar to those described for RT-qPCR. Specific primers utilized for ChIP-qPCR are meticulously outlined in the Supplementary Table S2.

### Dual-luciferase reporter gene assay

PDAC cells were suspended at a concentration of 2 × 10^4 cells/0.1 ml and seeded into a 96-well plate. Subsequently, they were transfected with reporter plasmids at a concentration of 100 ng/well, along with the corresponding expression plasmids, following the protocols described in the cell culture and transfection section. A pRL-TK vector (Promega, USA), encoding Renilla luciferase, was employed as an internal control to assess transfection efficiency. Luciferase activities in each well were quantitatively measured using the Dual-Luciferase Reporter Assay System (Promega, USA).

### Flow cytometry analysis of the mouse allograft samples

The tumors were dissected into smaller fragments and incubated in DMEM supplemented with 2 mg/ml of collagenase (Sigma, USA) for 1 h at 37 °C to facilitate cell dissociation. Subsequently, a 70 μm nylon mesh filter was utilized to separate the digested cells from any residual tissue fragments. The isolated cells were then resuspended in PBS containing 2% BSA and co-labeled with the specified antibodies (Detailed information regarding the antibodies is provided in the Supplementary Table). After a 15-minute incubation period with the antibodies, the cells were washed with PBS and subjected to flow cytometry for analysis.

### Immunohistochemistry (IHC)

Immunohistochemical analysis was performed with antibodies specific for Bap1 (working dilution 1:500, Proteintech), Pd-l1 (working dilution 1:1000, Proteintech). The scoring methodology entailed a comprehensive consideration of both the intensity of staining (graded on a scale of 0 to 3) and the positive rate (ranging from 0 to 100% of positively stained cells). Subsequently, these two components were multiplied to derive a comprehensive IHC score, providing a quantitative measure of protein expression levels.

### TIMER analysis

In this study, the web server TIMER database (https://cistrome.shinyapps.io/timer/) was utilized for detecting the relationship of BAP1 expression with multiple immune cells in PDAC. TIMER web server applies a deconvolution method to infer the abundance of tumor-infiltrating immune cells and spearman correlation to investigate the relationship of BAP1 expression with multiple immune cells in PDAC.

### Statistical analysis

The differential expressed genes (DEGs) in TCGA pancreatic cancer dataset was defined by DESeq2 (https://bioconductor.org/packages/release/bioc/html/DESeq2.html). Genes with *P* < 0.05 were identified as differentially expressed between two groups. Then, the clusterprofilerv4.0Rpackage and gene signatures from Msigdb were used for KEGG and GSEA.

GraphPad Prism 9 software (GradPad Software, Inc) was used for all statistical analyses. Statistical significance was assessed using the Student’s t-test, and one or two-way ANOVA, followed by Tukey’s multiple comparison tests. Only P values less than 0.05 were considered significant. All the values are expressed as the mean ± SD.

## Results

### BAP1 deficiency resulted in the resistance to immunotherapy

**Fig. 1:**
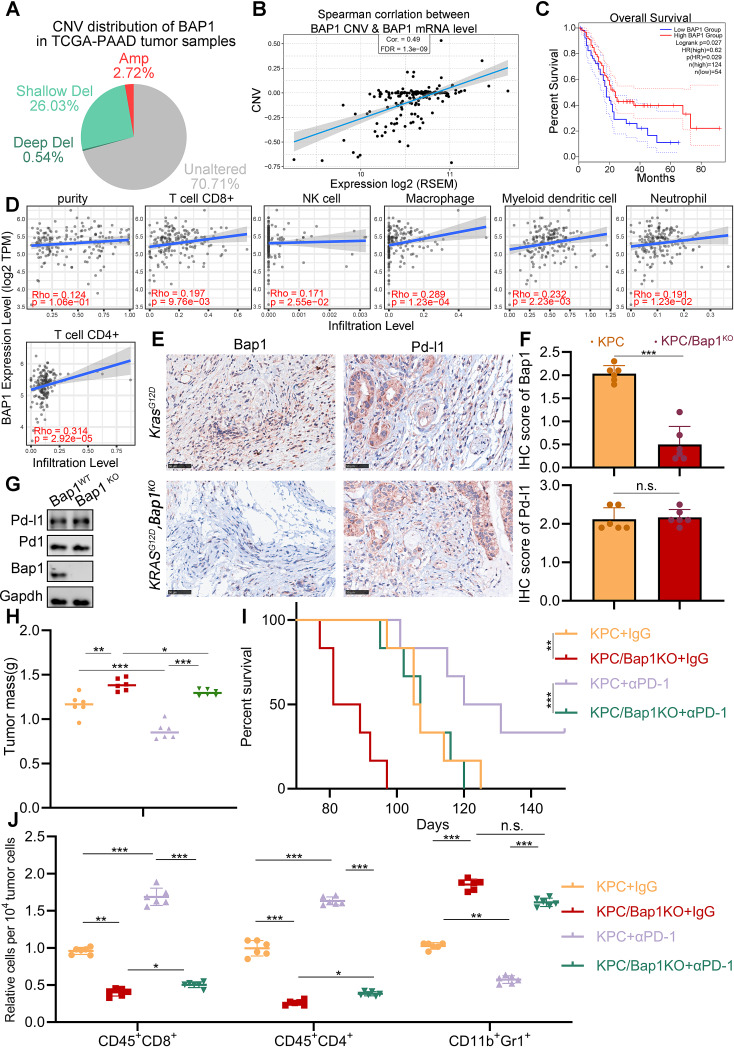
BAP1 deficiency resulted in the resistance to immunotherapy. **(A)** A pie chart illustrating the copy number alterations of BAP1 in the TCGA-PAAD dataset. **(B)** A scatter plot depicting the correlation between BAP1 copy number alterations and mRNA levels in the TCGA-PAAD dataset. **(C)** Survival analysis of patients grouped based on high and low expression of BAP1 in TCGA-PAAD dataset. **(D)** The analysis investigated the correlation between BAP1 expression and the indicated tumor infiltrated immune cells within the PAAD tumors using the Tumor Immune Estimation Resource (TIMER, cistrome.shinyapps.io/timer). **(E-F)** Representative images **(E)** and IHC score **(D)** of Bap1 and Pd-l1 in KPC/KPC; Bap1^KO^ mice. *n* = 6, two-tailed unpaired Student’s t-test. **(G)** Western blot analysis of indicated genes of the KPC mice. **(H)** The comparison of pancreas mass in the specified groups. *n* = 6,two-tailed unpaired t test. (n.s. not significant, * *P* < 0.05, *** *P* < 0.001). **(I)** Survival analysis of the KPC/KPC; Bap1^KO^ mice in specified groups (*n* = 6, ** *P* < 0.01). **(J)** FACS analysis of tumor infiltrated CD45 + CD8 + T cells, CD45 + CD4 + T cells, and CD11b + Gr1 + myeloid cells in indicated groups (*n* = 6, n.s. not significant, * *P* < 0.05, *** *P* < 0.001)

Diverging from its status in melanoma and cholangiocarcinoma, where the primary genomic alteration of BAP1 occurs as a mutation, the predominant genomic alteration of BAP1 in pancreatic cancer is copy number loss (shallow deletion 26.03%, deep deletion 0.54%) (Fig. 1A). The reduction in the copy number of the *BAP1* gene leads to a significant downregulation of BAP1 expression at the mRNA level (Fig. 1B) and is notably associated with poorer overall survival among patients (Fig. 1C) in the Cancer Genome Atlas (TCGA) PAAD dataset. BAP1 has been reported to regulate antitumor immunity in clear cell renal cell carcinoma, although the specific mechanism remains undisclosed [12]. Intrigued by this finding, we sought to investigate whether BAP1 similarly regulates cancer immunity, using pancreatic cancer as our working model.

Interestingly, we observed a positive correlation between BAP1 expression and the level of tumor-infiltrating immune cells, including CD8^+^ T cells, NK cells, CD4^+^ T cells, macrophage, neutrophil and dendritic cells, but not Myeloid-derived suppressor cells (Fig. 1D, Supplementary Fig. S1A) in pancreatic cancer tissues [19]. To validate this phenomenon, we generated control KPC (*KrasG12D/+; LSLTrp53R172H/+; Pdx-1-Cre*) mice and Bap1 knockout KPC mice, and found that Bap1 expression has minimal effect on the expression of Pd-l1 (Fig. 1E-G). However, a significantly increased tumor burdens in Bap1 knockout KPC mice was observed (Fig. 1H), and mice harboring Bap1 deletion exhibited poorer survival compared to those control KPC mice(Fig. 1I). Importantly, data on tumor mass and mouse survival both indicate that Bap1 knockout tumors exhibited an unresponsive status to immune checkpoint blockade therapy, such as anti-PD1 antibody (Fig. 1H-I). FACS analysis of the tumors validated this hypothesis. Bap1 knockout resulted in a notably decrease in the tumor infiltration of CD45^+^CD8^+^ T cells and CD45^+^CD4^+^ T cells, while increased the infiltration of repressive CD11b + Gr1 + myeloid cells (Fig. 1J). Furthermore, anti-PD1 therapy failed to elevate the infiltration of CD45^+^CD8^+^ and CD45^+^CD4^+^ T cells in Bap1 knockout tumors, as observed in control tumors (Fig. 1J). Moreover, we generated control and Bap1 knockout KPC (*KrasG12D/+; LSLTrp53R172H/+; Pdx-1-Cre*) mouse pancreatic cancer cell lines (Fig. 1E) and established the mouse syngeneic subcutaneous pancreatic cancer model (Supplementary Fig. S1B-C). The subcutaneous pancreatic cancer model also confirmed the above results (Supplementary Fig. S1D-F). Thus, data above suggest that BAP1 loss may lead to resistance to immunotherapy in pancreatic cancer.

We subsequently examined whether BAP1 regulates the expression of PD-L1 in pancreatic cancer cells. However, the knockout or overexpression of BAP1 did not significantly affect the expression of PD-L1 in the pancreatic cancer cell line (PANC-1), renal cancer cell line (OS-RC-2) and mesothelioma cell line (H2810) (Supplementary Fig. S1G-H). This suggests that BAP1 might not regulate tumor immunity by affecting the expression of PD-L1.

### BAP1 represses the transcriptional activity of HSF1

**Fig. 2:**
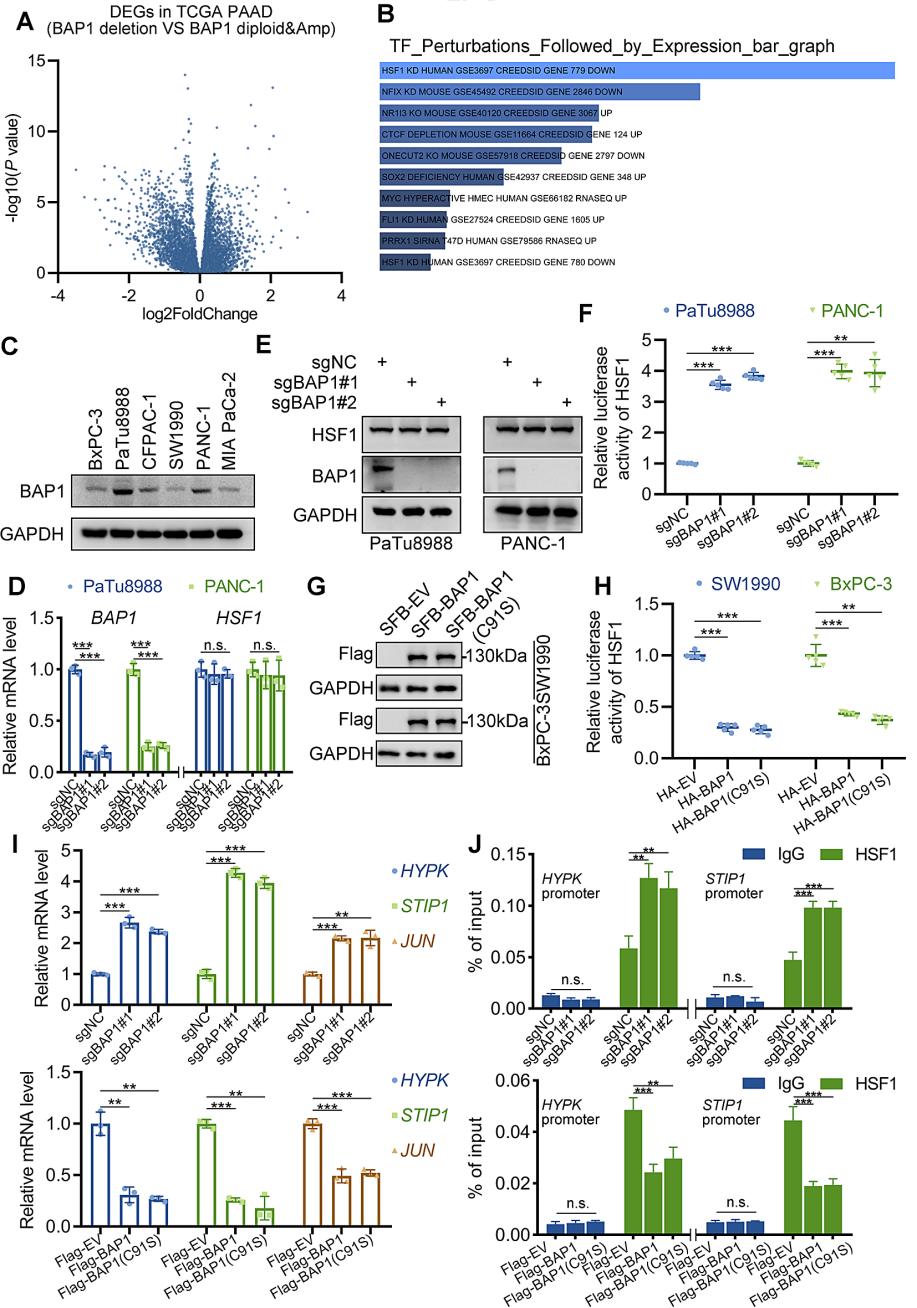
BAP1 represses the transcriptional activity of HSF1. **(A)** The volcano plot of differentially expressed genes between BAP1 deletion patients and BAP1 diploid & amplificated patients in TCGA-PAAD dataset. **(B)** The bar plot of the transcription factor enrich analysis of BAP1 downregulated genes. **(C)** The expression of BAP1 were examined via western blot in the indicated cell lines. **(D-E)** Cells infected with indicated sgRNAs and were screened using puromycin for 48 h, and were harvested for RT-qPCR analysis (*n* = 3, n.s. not significant, *** *P* < 0.001) (D) and western blot (E). **(F)** Cells infected with indicated plasmids and were harvested for western blot. **(G-H)** Luciferase reporter activities of HSF1 were detected in cells with indicated conditions (*n* = 5, ** *P* < 0.01, *** *P* < 0.001). **(I)** RT-qPCR analysis of indicated genes under conditions of BAP1 knock-out or overexpression (*n* = 3, ** *P* < 0.01, *** *P* < 0.001). **(J)** ChIP-qPCR analysis of HSF1 on the promoter of indicated genes under conditions of BAP1 knock-out or overexpression (*n* = 3, n.s. not significant, ** *P* < 0.01, *** *P* < 0.001)

BAP1 primarily acts as a transcriptional regulator through its control of H2A-Ub levels or interaction with other DNA-binding proteins [9, 20]. To delve deeper into the mechanism by which BAP1 regulates pancreatic cancer’s response to immunotherapy, we analyzed transcriptome data from the TCGA-PAAD dataset, comparing patients with BAP1 deletion to those with diploid or amplified BAP1 expression (Fig. 2A). We conducted reverse transcription factor enrichment analysis on significantly upregulated genes in patients with BAP1 deletion, and noticed that the downstream genes of the Heat Shock Transcription Factor 1 (HSF1) is significantly upregulated in BAP1 deletion patients (Fig. 2B). Recognized as a master regulator in the process of stress response, HSF1 and its signaling were also found to be significantly correlated with MHC-I machinery activation, tumor infiltrating T cells and check point blockade response in cancer patients [17]. We hypothesized that BAP1 likely regulates the immune therapy response by modulating the activity of HSF1. Therefore, we verified whether BAP1 affects the activity of HSF1. We generated BAP1 knock-out PDAC cells in PaTu8988 and PANC-1 cells, which exhibit relatively high BAP1 expression compared to other commonly used cell lines in our lab inventory (Fig. 2C-E). Interestingly, we found that BAP1 knock-out did not affect the mRNA or protein level of HSF1 (Fig. 2D-E). However, the luciferase report gene assay indicated that the knock-out of BAP1 notably upregulates the transcriptional activity of HSF1 (Fig. 2F). This phenomenon was further verified when we overexpressed BAP1 in SW1990 and BxPC-3 cells, cell lines with relatively low BAP1 expression (Fig. 2G). Notably, the overexpression of BAP1 enzymatic inactive mutant (BAP1(C91S)) exhibited equivalent repression on HSF1 activity compared to the overexpression of wild type (WT) BAP1 (Fig. 2G-H). Furthermore, we observed consistent phenomena, when we performed RT-qPCR and HSF1 ChIP-qPCR analysis with several classical downstream genes of HSF1 (*HYPK*,* STIP1* and *JUN*) (Fig. 2I-J). All these data indicated that BAP1 represses HSF1 transcriptional activity in a manner independent on its enzymatic activity.

We performed RNA-seq analysis in control or HSF1 knock-down (KD) PaTu8988 cells (Supplementary Fig. S2A). As the data above has confirmed that BAP1 inhibits the transcriptional activity of HSF1, we selected genes that are downregulated by HSF1 KD for enrichment analysis. Further analysis of the transcriptome data indicated that TNF signaling pathway was dramatically enriched in genes downregulated by HSF1 KD (Supplementary Fig. S2B). Furthermore, RT-qPCR and HSF1 ChIP-qPCR analysis with several classical genes (*ATF4* and *CREB5*) of TNF signaling pathway indicated that BAP1 represses their expression via HSF1 (Supplementary Fig. S2C-D). These data indicate that BAP1 might regulates tumor immunity via HSF1-TNF signaling.

### BAP1 interacts with the N-terminal of HSF1

**Fig. 3:**
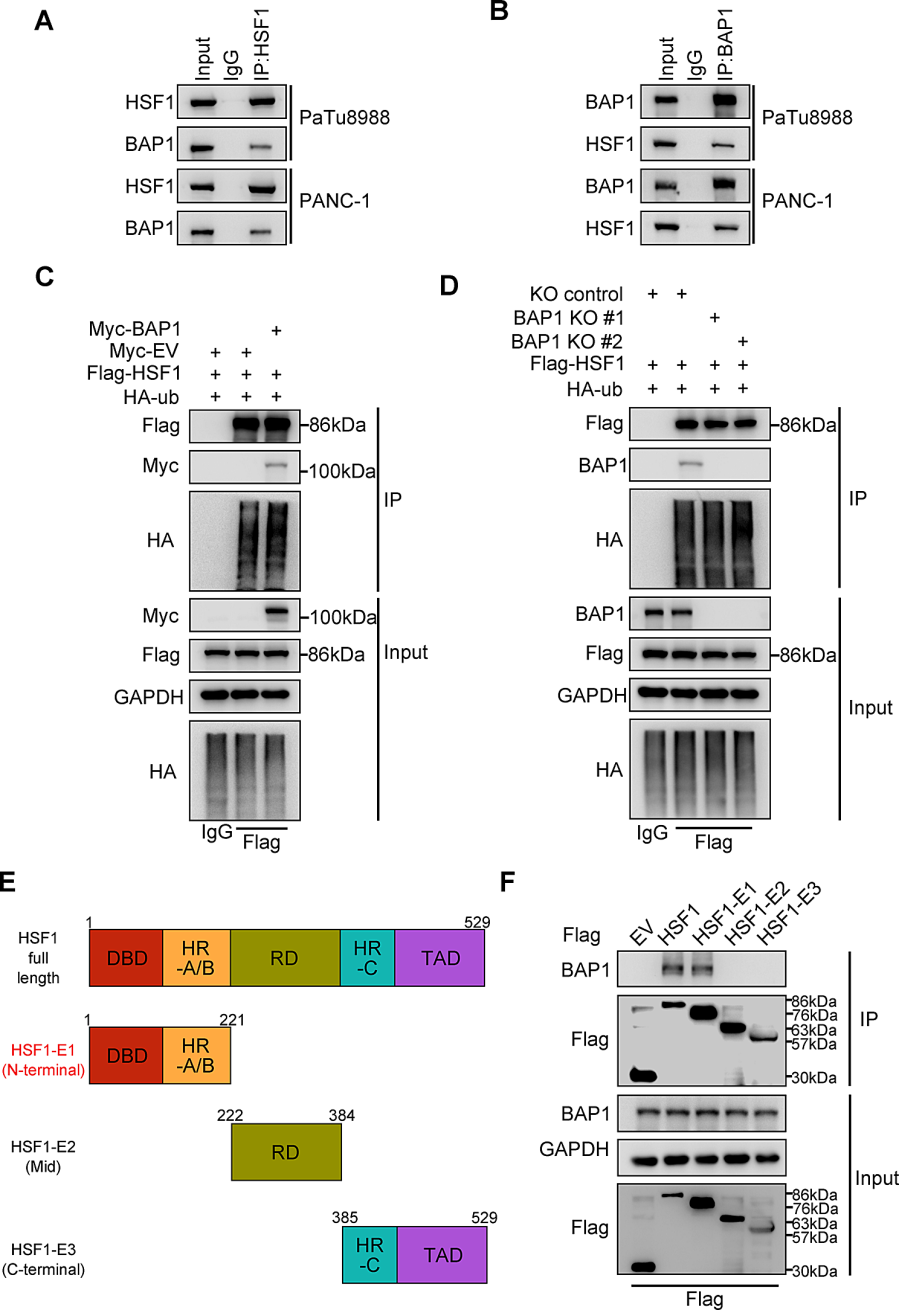
BAP1 interacts with the N-terminal of HSF1. **(A-B)** Endogenous Co-IP of the indicated proteins in PANC-1 and PaTu8988 cells. **(C-D)** Co-IP assay to detect the ubiquitination level of HSF1 in the indicated status of 293T cells. **(E)** Schematic depicting the domain structure and the truncation constructs. **(F)** 293T cells were transfected with indicated constructs and then were harvested for Co-IP and WB analysis

Given that BAP1 modulates the transcriptional activity of HSF1 without impacting its expression, we sought to investigate whether BAP1 exerts its influence on HSF1 activity through post-transcriptional mechanisms. The endogenous reciprocal co-immunoprecipitation (Co-IP) of BAP1 and HSF1 in PANC-1 and PaTu8988 cells demonstrated the protein-protein interaction (PPI) between the two (Fig. 3A-B). Interestingly, neither the overexpression nor the knockout of BAP1 affected the ubiquitination level of HSF1 (Fig. 3C-D), which is consistent with our previous observation that both wild-type (WT) and enzymatically inactive BAP1 were capable of repressing HSF1 (Fig. 2G and I). To delve deeper into how BAP1 suppresses HSF1 activity through protein-protein interaction (PPI) with HSF1, we proceeded to construct truncations of HSF1 based on its major functional domains (Fig. 3E). The subsequent Co-IP assay revealed that the N-terminal of HSF1 was pivotal for the formation of the BAP1-HSF1 complex (Fig. 3F).

### BAP1 facilitates HSF1 monomerization by augmenting HSP70-HSF1 interaction

**Fig. 4:**
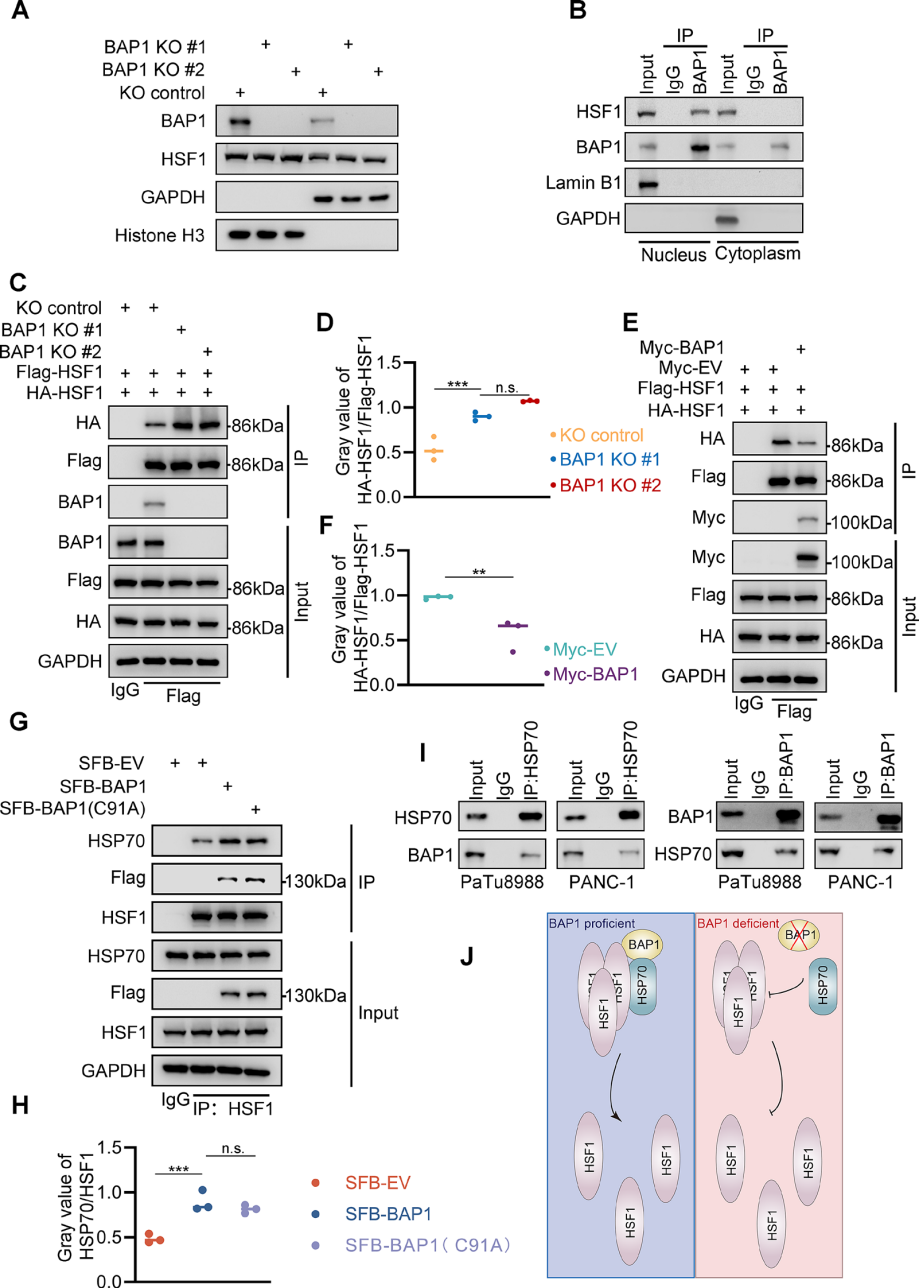
BAP1 facilitates HSF1 monomerization by augmenting HSP70-HSF1 interaction. **(A)** Western blot analysis was performed on cytosolic and nuclear fractions of PANC-1 cells infected with lentivirus expressing specified sgRNAs, followed by 48 h of puromycin selection. **(B)** Co-IP analysis of indicated proteins were performed on cytosolic and nuclear fractions of PANC-1 cells. **(C-D)** BAP1 stably knock-out PANC-1 cells were transfected with indicated constructs for 48 h and were harvested for Co-IP analysis(C) and quantification (D; *n* = 3 biologically independent samples) of gray value of HA-HSF1/Flag-HSF1. **(E-F)** Representative Co-IP analysis of the expression of indicated proteins in PANC-1 cells with transfected with indicated constructs (E) and quantification (F; *n* = 3 biologically independent samples) of gray value of HA-HSF1/Flag-HSF1. **(G-H)** PANC-1 cells were transfected with indicated constructs for 48 h and were harvested for Co-IP analysis(G) and quantification of gray value of HSP70/ HSF1. **(I)** Endogenous Co-IP of the indicated proteins in PANC-1 and PaTu8988 cells. **(J)** In the presence of proficient BAP1, BAP1 facilitates the recruitment of HSP70 by trimerized HSF1, thus promoting the dissociation of trimerized HSF1 into inactive monomeric HSF1. In the absence of BAP1, the recruitment of HSP70 by HSF1 is reduced, resulting in decreased dissociation of HSF1 and increased activity

The N-terminal of HSF1 contains the HR-A/B regions and DNA binding domain (DBD), which are responsible for HSF1 trimerization and DNA occupation, respectively. Upon receiving activation signals, HSF1 undergoes trimerization and translocate into the nucleus, thereby acquiring the ability and conditions necessary for DNA binding. However, the knockout of BAP1 does not affect the nuclear localization of HSF1 (Fig. 4A). Interestingly, the interaction between BAP1 and HSF1 exclusively occurs in the nucleus (Fig. 4B). Therefore, we attempted to determine whether BAP1 affects the oligomerization of HSF1 via the interaction with HSF1. Consistent with our hypothesis, the knockout of BAP1 significantly increases the interaction strength between the ectopically expressed HSF1 with different tags (Fig. 4C-D, Supplementary Fig. S3A-B), and the overexpression of BAP1 also attenuates this interaction (Fig. 4E-F, Supplementary Fig. S3C-D) in PANC-1 and PaTu8988 cells.

HSP70, the pivotal regulator of HSF1, not only interacts with the TAD domain to influence its transcriptional activity but also interacts with the HR-B domain, facilitating the transition of HSF1 from its trimeric form to monomeric state [21]. We were interested whether BAP1 is involved in this process. Co-IP assay indicated that both the WT and C91A mutant BAP1 similarly enhanced the interaction between HSP70 and HSF1 (Fig. 4G-H). Endogenous reciprocal co-immunoprecipitation (Co-IP) of BAP1 and HSP70 indicated the protein-protein interaction between the them (Fig. 4I), indicating BAP1 might recruit HSP70 to HSF1 trimer to promote HSF1 monomerization. Therefore, our data suggest a model that BAP1 likely inhibits the trimerization and transcriptional activity of HSF1 by enhancing the interaction between HSP70 and HSF1 (Fig. 4J).

### BAP1 inhibits the formation of SIRT1-HSF1 complex via competitive interaction

**Fig. 5:**
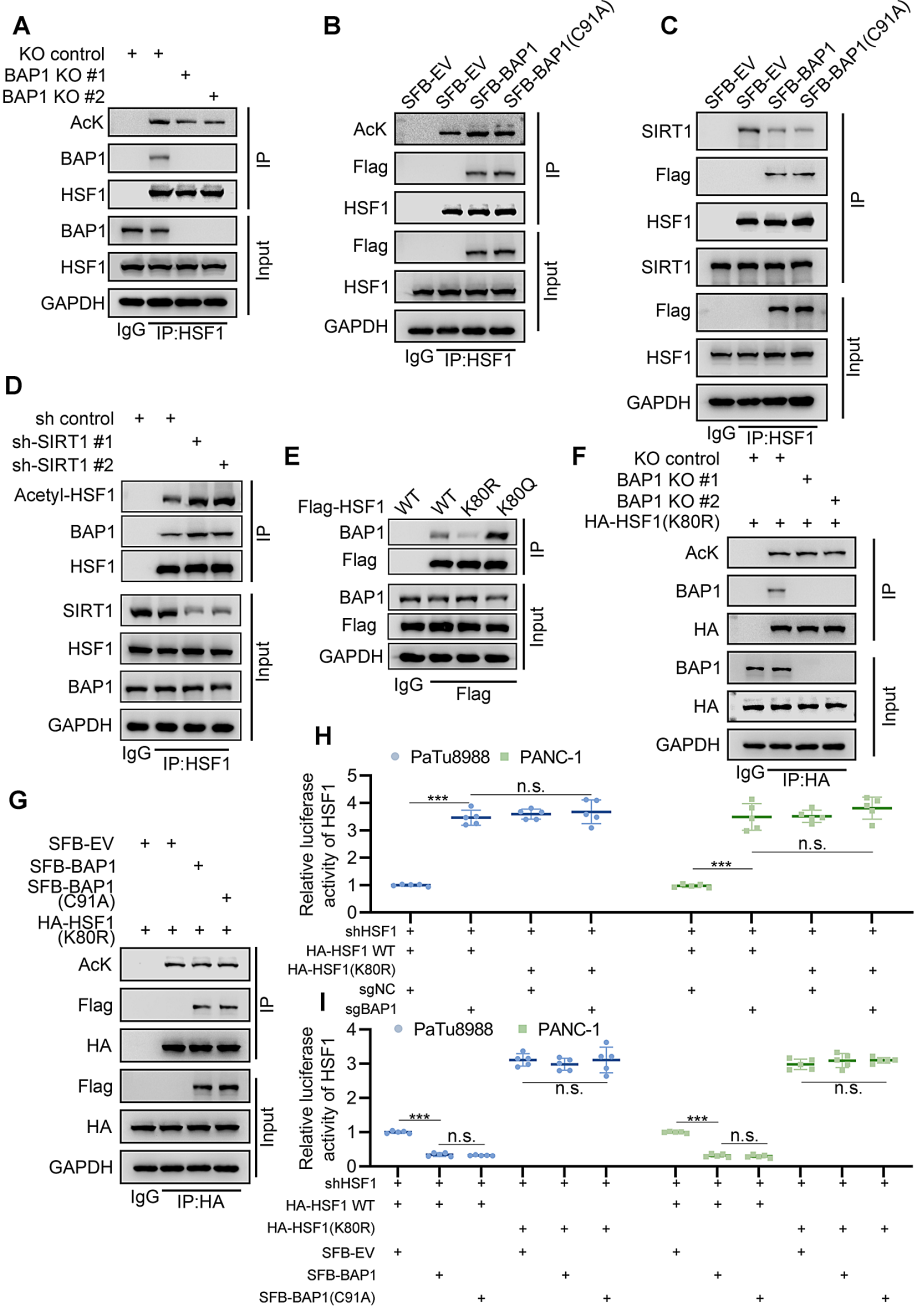
BAP1 inhibits the formation of SIRT1-HSF1 complex via competitive interaction. **(A)** PANC-1 cells were infected with lentivirus expressing specified sgRNAs and subsequently subjected to Co-IP analysis after 48 h of puromycin selection. **(B-C)** PANC-1 cells were transfected with indicated constructs and were harvested for Co-IP assay. **(D)** PANC-1 cells were infected with lentivirus expressing specified shRNAs and subsequently subjected to Co-IP analysis after 48 h of puromycin selection. **(E)** 293T cells were transfected with indicated constructs and were harvested for Co-IP. **(F)** BAP1 stably knock-out PANC-1 cells were transfected with indicated constructs for 48 h and were harvested for Co-IP analysis. **(G)** 293T cells were transfected with indicated constructs and were harvested for Co-IP. **(H-I)** Luciferase reporter activities of HSF1 were detected in PANC-1 and PaTu8988 cells under indicated conditions (*n* = 5, n.s. not significant, *** *P* < 0.001)

Despite BAP1 inhibiting the trimerization of HSF1 by enhancing the interaction between HSP70 and HSF1, the BAP1-HSF1 interaction remains unaffected by HSP70 (Fig. 4G), suggesting the existence of a potential mechanism for BAP1 to interact with HSF1 and subsequently recruit HSP70 to monomerize HSF1. Given that SIRT1 mediated lysine deacetylation in the DNA-binding domain (DBD) of HSF1 is crucial for the dissociation of HSF1 trimers from DNA [22], we were interested in investigating whether BAP1 is involved in this process. Notably, the knockout of BAP1 resulted in the suppression of HSF1 acetylation (Fig. 5A, Supplementary Fig. S3E), while the expression of either WT or C91A mutated BAP1 elevated the lysine acetylation level of HSF1 (Fig. 5B, Supplementary Fig. S3F). More importantly, both wild-type (WT) and C91A mutated BAP1 attenuated the HSF1-SIRT1 interaction (Fig. 5C, Supplementary Fig. S3G), and SIRT1 knockdown resulted in an enhanced binding between HSF1 and BAP1 (Fig. 5D, Supplementary Fig. S3H), indicating that BAP1 and SIRT1 might compete with each other for binding to HSF1.

Considering SIRT1 promotes HSF1 activity via the deacetylation at K80 of HSF1 [22], We constructed the acetylation resistant mutant (HSF1(K80R)) and acetylation mimic mutant (HSF1(K80Q)) to further investigate how HSF1-K80 acetylation influence HSF1-BAP1. Co-IP assay indicated that BAP1 exhibited remarkably enhanced interaction with HSF1(K80Q) mutant compared to WT HSF1, while the acetylation resistant mutation (HSF1(K80R)) almost abolished HSF1-BAP1 interaction (Fig. 5E, Supplementary Fig. S3I). Similarly, the knockout or overexpression of BAP1 no longer affects the acetylation levels of the HSF1(K80R) mutant (Fig. 5F-G). We also observed consistent phenomena in the luciferase report gene assay. The knockout or overexpression of BAP1 could only affect the transcriptional activity of WT HSF1, but not the HSF1-K80R mutant (Fig. 5H-I). Therefore, our data indicated that BAP1 competes with SIRT1 to interact with K80-acetylated HSF1, promoting its monomerization and dissociation from DNA.

### SIRT1 inhibition enhances immunotherapy efficacy in BAP1 deficient PDAC

**Fig. 6:**
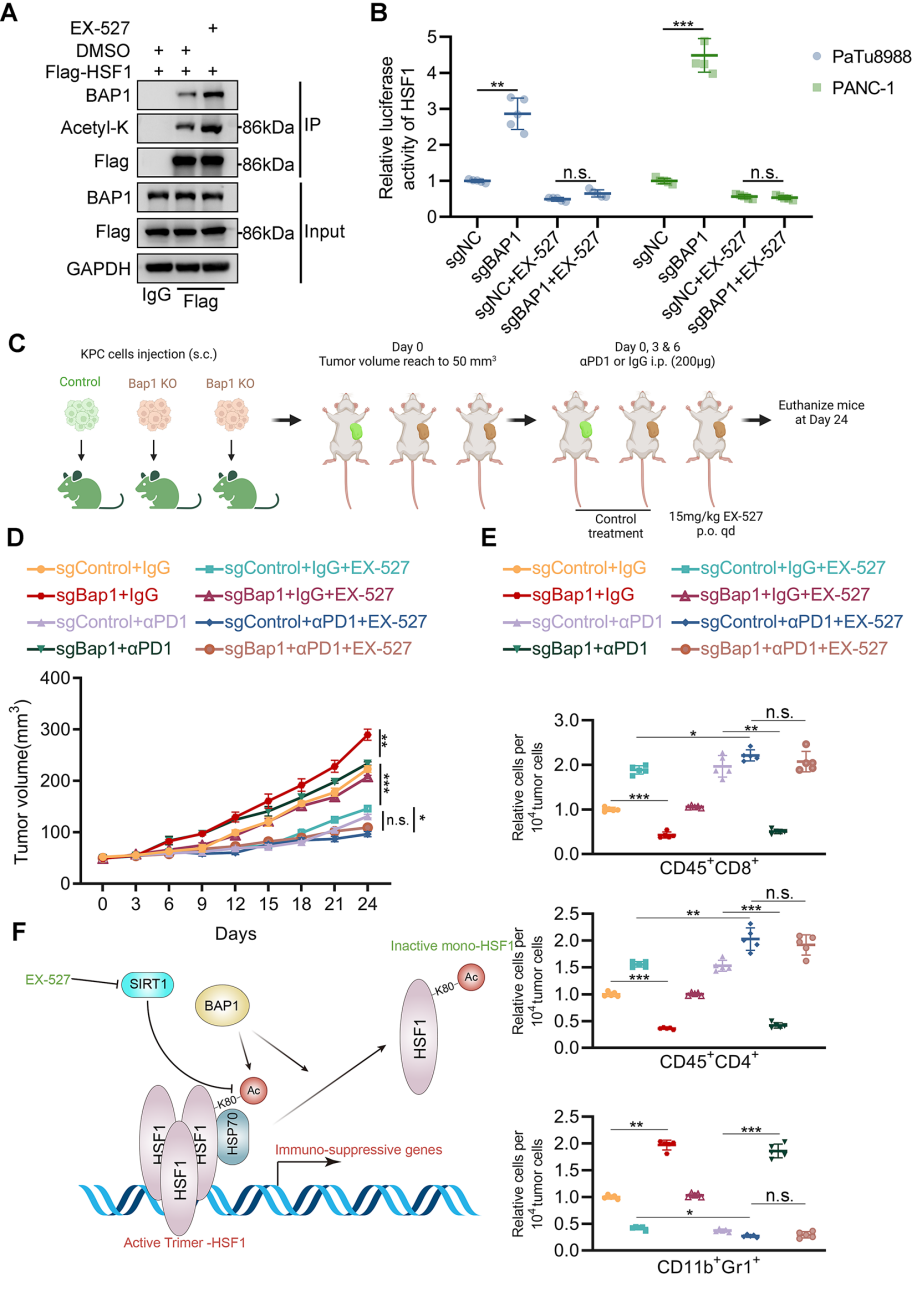
SIRT1 inhibition enhances immunotherapy efficacy in BAP1 deficient PDAC. **(A)** PANC-1 cells were transfected with indicated constructs for 24 h and were treated with control treatment or 200nM EX-527 for 36 h and were harvested for Co-IP. **(B)** BAP1 stably knock-out PANC-1 cells were harvested for the luciferase reporter assay for HSF1 after treated with indicated drugs for 36 h. **(C)** Diagrams depicting the process of constructing syngeneic xenograft models. **(D)** Line chart illustrating the progression of tumor growth in the specified groups (*n* = 5, n.s. not significant, ** *P* < 0.01, *** *P* < 0.001). **(E)** FACS analysis of tumor infiltrated CD45 + CD8 + T cells, CD45 + CD4 + T cells, and CD11b + Gr1 + myeloid cells in indicated groups (*n* = 5, n.s. not significant, ** *P* < 0.01, *** *P* < 0.001). **(F)** A hypothetical model illustrating BAP1 repressing HSF1 chromatin occupation via competitive interaction with K80 acetylated HSF1, countering SIRT1-mediated deacetylation and activation of HSF1

After observing the antagonistic relationship between BAP1 and SIRT1, particularly in their convergence on the K80 acetylation of HSF1, we became intrigued by the potential efficacy of combining SIRT1 inhibition with ICB therapy in models of pancreatic cancer characterized by BAP1 deletion. Co-immunoprecipitation assays indicated that treatment with the SIRT1 inhibitor, EX-527, notably augmented the level of acetylated lysine of HFS1, as well as the interaction between HSF1 and BAP1 (Fig. 6A). Additionally, EX-527 treatment attenuated the upregulation of HSF1 activity induced by BAP1 deletion (Fig. 6B).

We further validated our hypothesis using KPC cells constructed syngeneic subcutaneous xenograft models (Fig. 6C). Mice accepted EX-527 combined with anti-PD-1 therapy exhibited less tumor burdens than those accepted EX-527 alone (Fig. 6D-E), indicating the synergistic effect between EX-527 and anti-PD-1 therapy. More importantly, treatment with EX-527 in combination with anti-PD-1 therapy, nearly abrogated the immunotherapeutic resistance conferred by BAP1 deficiency (Fig. 6D-E).

In summary, our study revealed the mechanism by which BAP1 inhibits HSF1 oligomerization by safeguarding HSF1-K80 acetylation, thereby suppressing HSF1 transcriptional activity (Fig. 6F). Additionally, it suggests that SIRT1 inhibition combined with ICB therapy may ameliorate the unresponsiveness of BAP1-deficient pancreatic cancer to single-agent immunotherapy.

## Discussion

BAP1 is ubiquitously expressed in the nucleus and cytoplasm. While its enzymatic activity requires nuclear localization, which allows it, along with associated proteins such as Additional sex combs-like 1 (ASXL1), 2, and 3, to form the polycomb repressive deubiquitinase (PR-DUB) complex, thereby regulating chromatin conformation and gene transcription [23, 24]. Besides, BAP1 also interacts with numerous DNA binding proteins to further regulate transcription, such as KMT2C, HCF-1 and FOXK1/2 [8, 25, 26]. Our current study identified an enzymatic activity independent function of BAP1, a co-repressor of HSF1. BAP1-HSF1 interaction inhibits the deacetylation of HSF1-K80 mediated by SIRT1, thereby promoting HSP70 mediated monomerization of HSF1 and its dissociation from target gene loci. Interestingly, BAP1 autodeubiquitination is crucial for its nuclear localization [24]. Theoretically, the enzymatic activity of BAP1 could affect its interaction with HSF1. However, our data indicated that the BAP1(C91S) mutant exhibited a comparable capacity to WT BAP1 in pulling down HSF1 in Co-IP assays, as well as a similar inhibitory effect on HSF1 activity in luciferase reporter assays. This may suggest that the binding between BAP1 and HSF1 could inhibit the ubiquitination of BAP1 mediated by UBE2O [24], or there may be other unexplored mechanisms at play. However, these interrogations do not undermine our conclusion that BAP1 inhibits HSF1 transcriptional activity independently of its enzymatic activity.

As previously stated, the significance of BAP1 in maintenance of genomic integrity, DNA damage repair, cellular metabolism, and cell death is relatively well-established [27–30]. However, its involvement in cancer immunity remains controversial. In malignancies like peritoneal mesothelioma, BAP1 inactivation was linked to increased tumor infiltrated immune cells and cell-attracting chemokines [31, 32]. While in uveal melanoma and ccRCC, BAP1 loss of expression is associated with immunosuppressive microenvironment [12, 33]. As previous mentioned, mutation or deletion of BAP1 may exhibit a correlation with the expression levels of PD-L1 in certain tumor types, such as malignant peritoneal/ pleural mesothelioma, thymic carcinoma and uveal melanoma [31, 34–36]. Furthermore, in the colon cancer cell line MC38, deletion of BAP1 results in decreased expression of PD-L1 [37]. However, our observations in this study suggested in BAP1 frequently altered malignancies, such as renal cancer, mesothelioma and pancreatic cancer, manipulating the expression of BAP1 have limited effect on the expression of PD-L1 of the tumor cells. These observations could potentially be attributed to inherent differences between in vitro and in vivo experimental setups, coupled with the diversity among various cell lines, highlighting the complexity of biological systems. However, pancreatic cancer is a type of cancer where the sensitivity to ICB treatment, as clinically proven, is not related to PD-L1 expression. Further investigation on the regulation of the immune microenvironment and related signaling pathways might be help to overcoming the current challenges in PDAC. In this study, we identified BAP1 regulates tumor immune microenvironment and the sensitivity to ICB treatment in a PD-L1 independent manner, and demonstrated a potential effective strategy to overcome the un-responsiveness to ICB therapy in BAP1 deficient pancreatic cancer [37].

Therapeutic strategies based on the biological function of BAP1 have been developed and evaluated for tumor patients harboring BAP1 deficiencies or alterations [38], such as histone deacetylase (HDAC) inhibitor, EZH2 inhibitor and PARP inhibitor [39–42]. However, the therapeutic outcomes have been less than satisfactory. Moreover, in the realm of immunotherapy, there are clues suggesting that the combination of ICB therapy with anti-CD38/anti-CD74 complexes or ICB therapy combined with CCR5 inhibitors may ameliorate the immune suppression caused by BAP1 deficiency [12, 32], while these strategies have not yet entered the clinical trial stage. Building upon the mechanism of BAP1-mediated inhibition of HSF1 through antagonizing SIRT1, we preliminarily validated the therapeutic effects of ICB therapy in combination with SIRT1 inhibitors in BAP1-deficient tumors in PDAC mouse model. Similarly, this treatment strategy also requires more robust validation.

HSF1 plays a critical role in protein homeostasis, DNA damage response, tumor migration, and metabolic processes [43–46]. Noteworthy, its regulatory role in tumor immunity is increasingly worthy of attention. HSF1 is observed to repress proinflammatory genes and cytokines [47], and inhibit the antigen presentation of the tumor cells [17, 48], thereby facilitating the immune surveillance evasion of the tumor. HSF1 was also suggested to regulate the expression of PD-L1 in breast cancer [49], but consistent observations have not been found in other cancers, including PDAC. However, manipulate HSF1 and its related signaling might be a promising strategy for immunotherapy sensitization.

Acetylation of HSF1 regulates its function in multiple ways. EP300-mediated acetylation on K208 and K298 of HSF1 stabilizes the HSF1 protein [50], while acetylation of K80 in the DNA-binding domain (DBD) of HSF1 impairs its affinity for DNA. SIRT1-mediated deacetylation of HSF1 counteracts this effect and preserves HSF1 activity [22]. Monomer-trimer transition is another critical mechanism that regulating HSF1 DNA binding [51]. HSP70 system is the major effector to monomerize HSF1 and promote HSF1’s detachment from DNA [51]. Our data indicate that BAP1 intricately regulates HSF1 through deep involvement in the aforementioned processes. BAP1-HSF1interaction impedes SIRT1 mediated deacetylation and recruit HSP70 to facilitate HSF1 monomerization.

Conclusively, our current study observed the immuno-suppressive role of BAP1 loss in PDAC, elucidated a previously undisclosed mechanism whereby BAP1 regulates cancer immunity through HSF1 repression, and provided a theoretical basis for combining SIRT1 inhibitors with ICB therapy for BAP1-deficient PDAC.

## Conclusion

In conclusion, our findings indicate that BAP1 exerts an inhibitory effect on HSF1 activation by engaging in competitive binding with SIRT1, ultimately culminating in immunosuppression. Collectively, the integration of SIRT1 inhibitors with ICB therapy emerges as a promising therapeutic avenue for addressing BAP1-deficient tumors.

## Electronic supplementary material

Below is the link to the electronic supplementary material.

Supplementary Material 1

Supplementary Material 2

Supplementary Material 3

Supplementary Material 4

Supplementary Material 5

Supplementary Material 6

Supplementary Material 7

Supplementary Material 8

## Data Availability

The datasets used and/or analyzed during the current study are available from the corresponding author (Zhiqiang Liu, drliuzq@hust.edu.cn) on reasonable request.

## Abbreviations

ChIP: Chromatin immunoprecipitation
qRT-PCR: Quantitative reverse; transcriptase polymerase chain reaction
TCGA: The Cancer Genome Atlas
RNA-seq: RNA sequencing
siRNAs: Small interfering RNAs
SDS-PAGE: Sodium dodecyl sulfate polyacrylamide gel electrophoresis
PBS: Phosphate-buffered saline
DMEM: Dulbecco’s Modified Eagle’s Medium
BAP1: BRCA1 associated protein-1
HSF1: Heat shock transcription Factor 1
FBS: Fetal bovine serum
KEGG: Kyoto Encyclopedia of Genes and Genomes
