# Supplementary figures and images for: BAP1 regulates HSF1 activity and cancer immunity in pancreatic cancer
Source: J Exp Clin Cancer Res. 2024 Sep 30;43:275. doi: 10.1186/s13046-024-03196-4 (PMC11441124; doi:10.1186/s13046-024-03196-4)

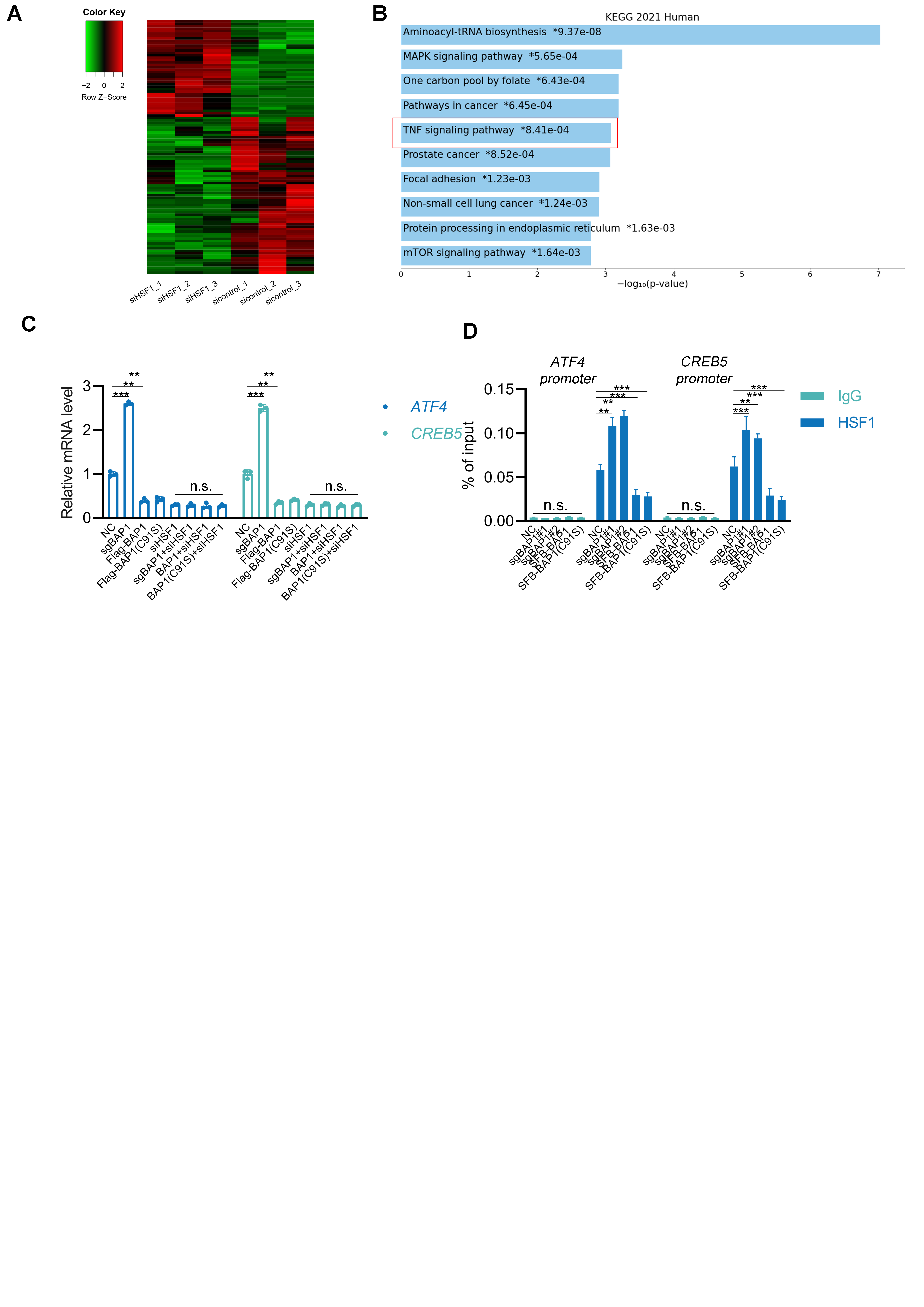

Supplement: Supplementary file 1 — Supplementary Material 1 [file 13046_2024_3196_MOESM1_ESM.tif]

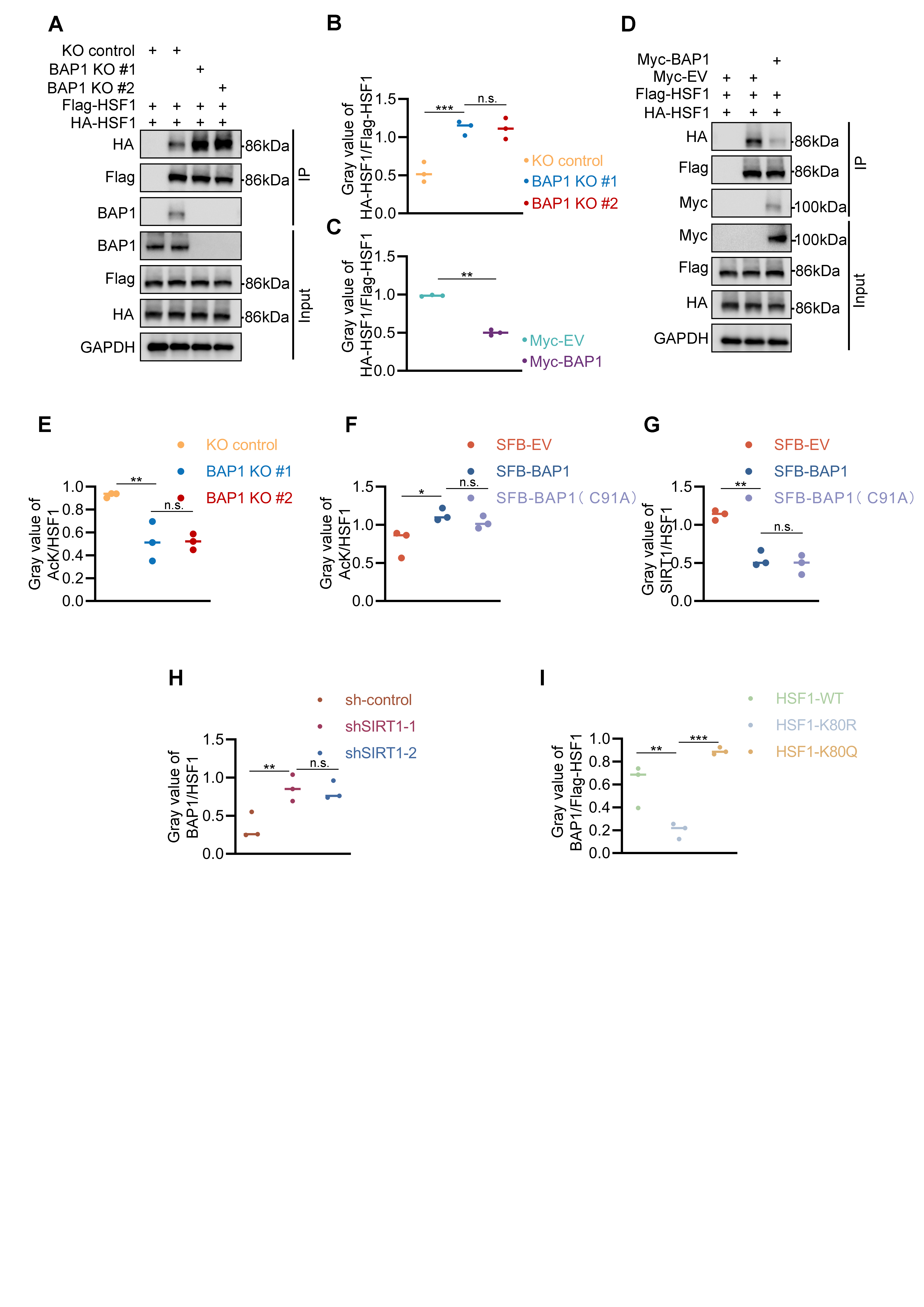

Supplement: Supplementary file 2 — Supplementary Material 2 [file 13046_2024_3196_MOESM2_ESM.tif]

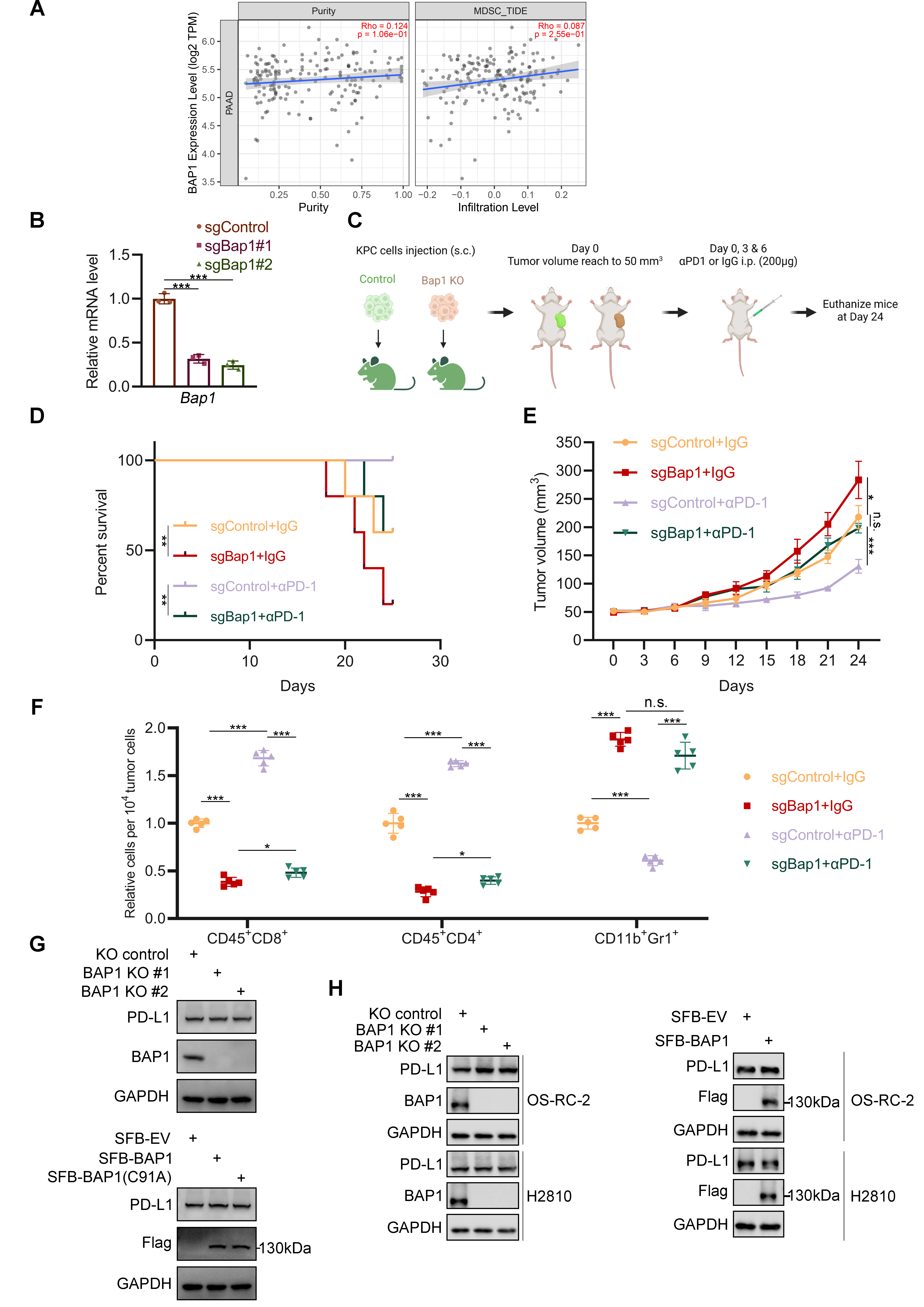

Supplement: Supplementary file 3 — Supplementary Material 3 [file 13046_2024_3196_MOESM3_ESM.tif]
