# Supplementary material for: BAP1 regulates HSF1 activity and cancer immunity in pancreatic cancer
Source: J Exp Clin Cancer Res. 2024 Sep 30;43:275. doi: 10.1186/s13046-024-03196-4 (PMC11441124; doi:10.1186/s13046-024-03196-4)
Supplement: Supplementary file 4 — Supplementary Material 4 [file 13046_2024_3196_MOESM4_ESM.docx]

**Supplementary materials for**

**BAP1 regulates HSF1 activity and cancer immunity in pancreatic cancer**

Weiwei Yuan, Qiyue Zhang, Yuhan Zhao, Wentao Xia, Shilin Yin, Xueyi Liang, Taoyu Chen, Gaofeng Li, Yanshen Liu, Zhiqiang Liu, Jinxi Huang

**The file includes:**

- Supplementary figures and figure legends
- Supplementary Table S1. Information of recombinant DNA
- Supplementary Table S2. Sequence of primers and gene specific shRNAs & SiRNAs
- Supplementary Table S3. Information of antibodies
- Supplementary Table S4. Information of chemicals and kits

**Supplementary figures and figure legends**

**
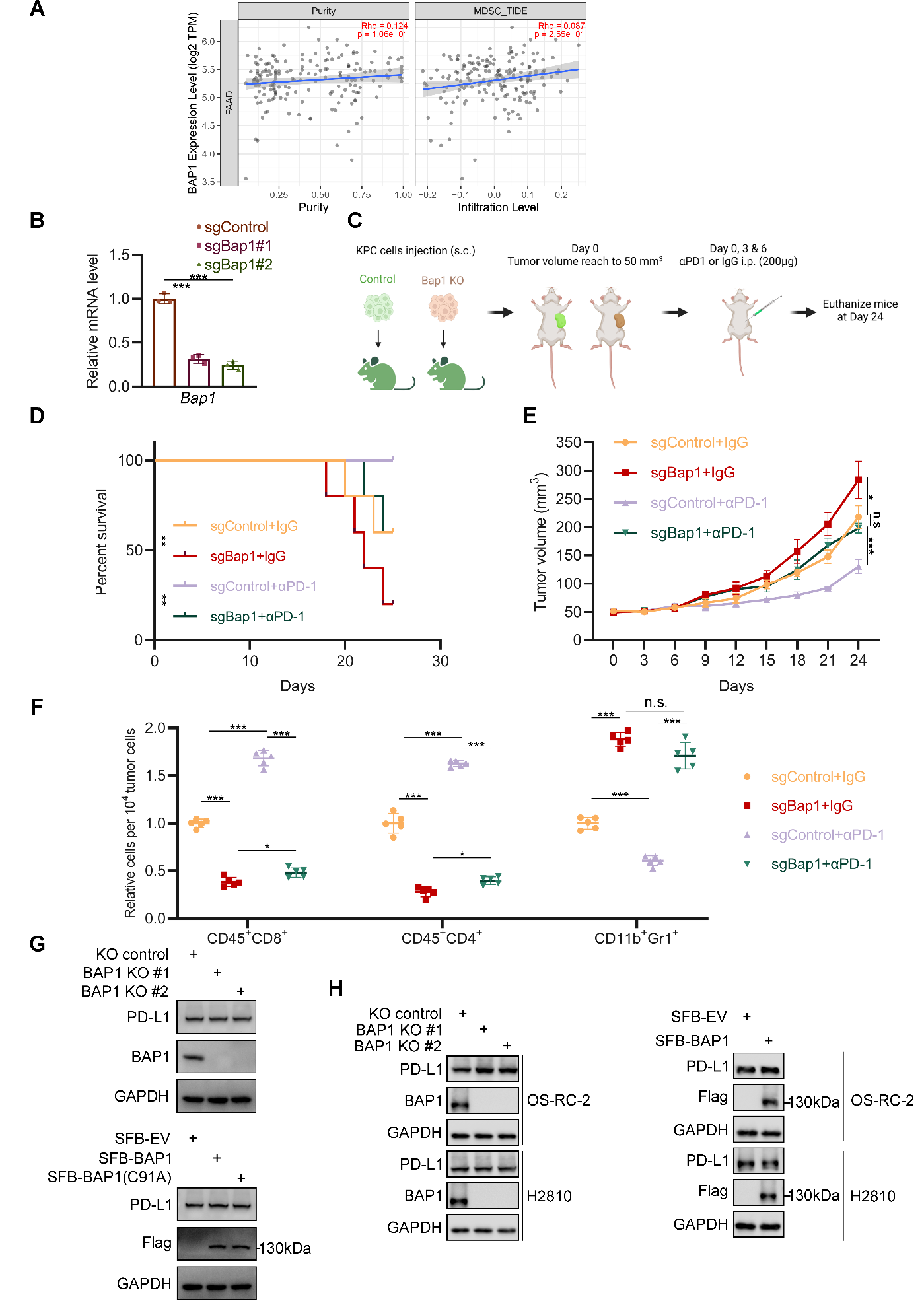
**

**Supplementary Figure S1 BAP1 deficiency resulted in the resistance to immunotherapy. (A)** The analysis investigated the correlation between BAP1 expression and the indicated tumor infiltrated immune cells within the PAAD tumors using the Tumor Immune Estimation Resource (TIMER, cistrome.shinyapps.io/timer). **(B)** KPC cells infected with indicated sgRNAs and were screened using puromycin for 48hrs, and were harvested for RT-qPCR analysis (n=3, *** *P* < 0.001). **(C)** Diagrams depicting the process of constructing syngeneic xenograft models. **(D)** Survival analysis of the tumor bearing mice in specified groups (n=5, ** *P* < 0.01). **(E)** Line chart illustrating the progression of tumor growth in the specified groups (n=5, n.s. not significant, * *P* < 0.05, *** *P* < 0.001). **(F)** FACS analysis of tumor infiltrated CD45+CD8+ T cells, CD45+CD4+ T cells, and CD11b+Gr1+ myeloid cells in indicated groups (n=5, n.s. not significant, * *P* < 0.05, *** *P* < 0.001). **(G)** PANC-1 cells were infected with lenti-virus expressing indicated sgRNAs, and were harvested for western blot analysis after 48hrs puromycin selection(above). PANC-1 cells were transfected with indicated plasmids for 36hrs and then were harvested for western blot analysis(below). **(H)** OS-RC-2 and H2810 cells were infected with lenti-virus expressing indicated sgRNAs, and were harvested for western blot analysis after 48hrs puromycin selection(left). OS-RC-2 and H2810 cells were transfected with indicated plasmids for 36hrs and then were harvested for western blot analysis(right).

**
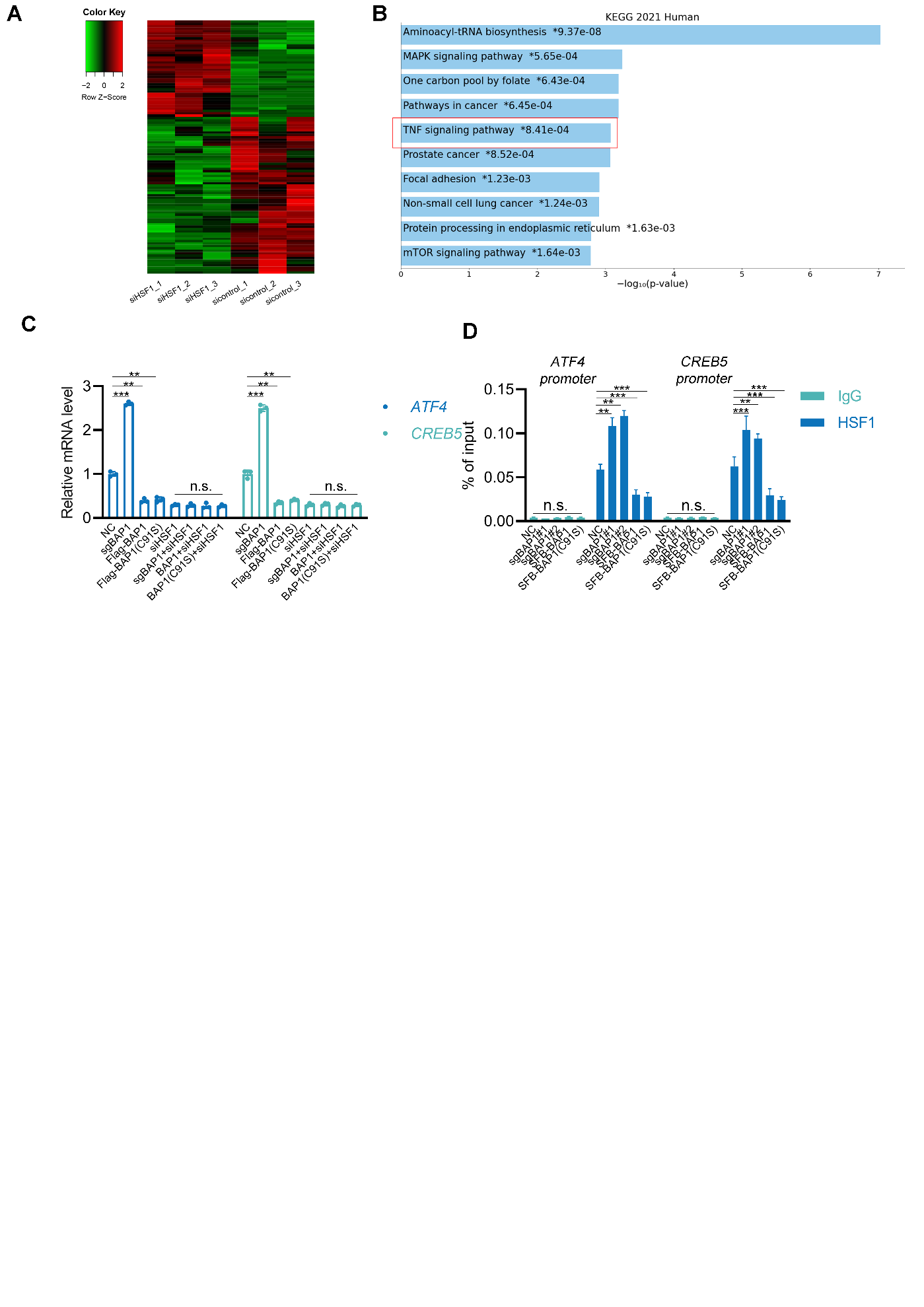
**

**Supplementary Figure S2 HSF1 regulates immunotherapy sensitization via TNF signaling pathway. (A)** Heatmap show the results of genome-wide analysis of RNA-seq of PaTu8988 cells with/without HSF1 KD. **(B)** Bar plot of the KEGG enrich analysis of HSF1 KD downregulated genes. **(C)** RT-qPCR analysis of indicated genes under conditions of BAP1 knock-out or overexpression with/without HSF1 KD (n=3, ** P < 0.01, *** P < 0.001). **(D)** ChIP-qPCR analysis of HSF1 on the promoter of indicated genes under conditions of BAP1 knock-out or overexpression (n=3, n.s. not significant, ** *P* < 0.01, *** *P* < 0.001).

**
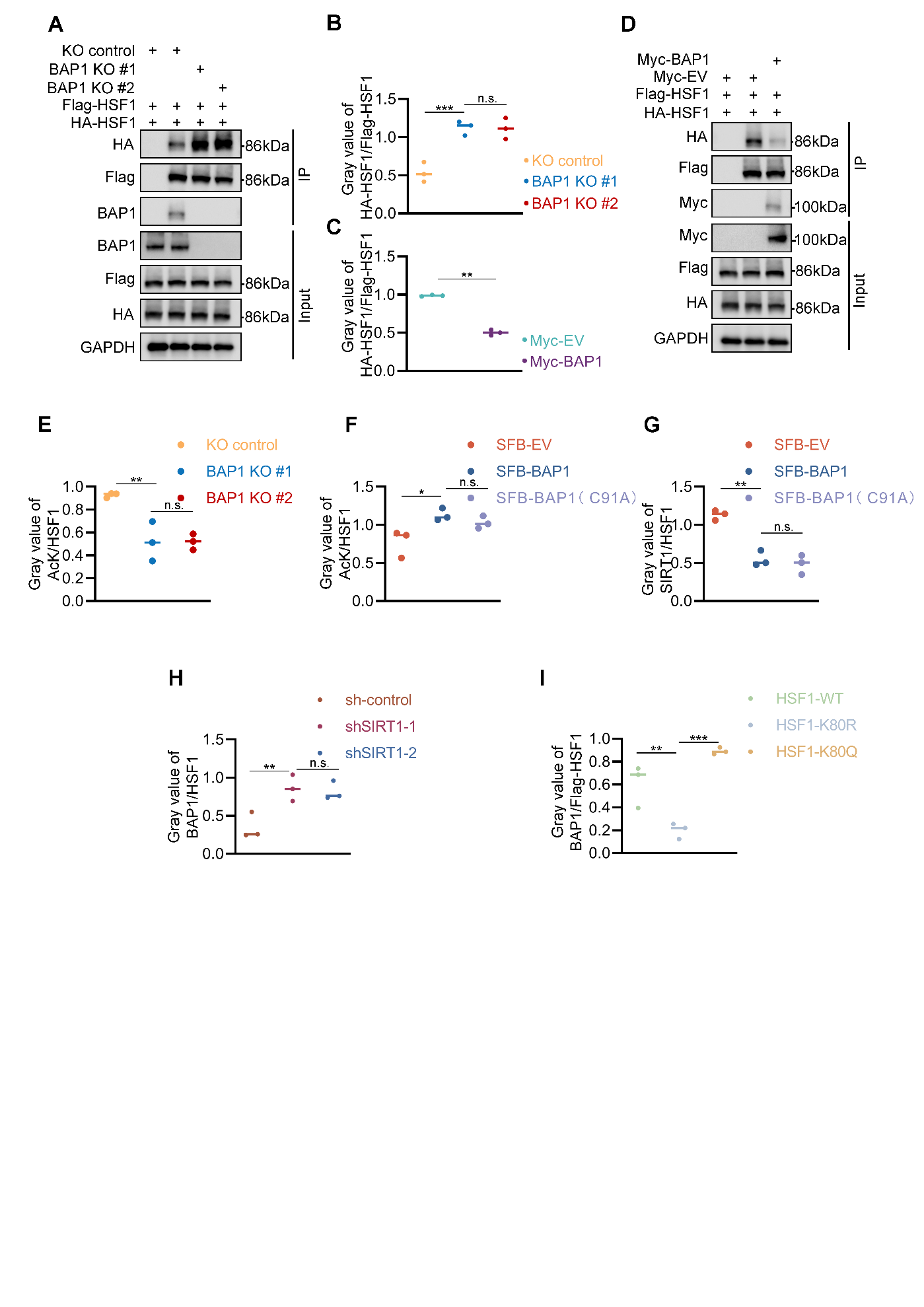
**

**Supplementary Figure S3 BAP1 facilitates HSF1 monomerization and inhibits the formation of SIRT1-HSF1 complex via competitive interaction. (A-B)** BAP1 stably knock-out PaTu8988 cells were transfected with indicated constructs for 48hrs and were harvested for Co-IP analysis(A) and quantification (B; n = 3 biologically independent samples) of gray value of HA-HSF1/Flag-HSF1. **(C-D)** Representative Co-IP analysis of the expression of indicated proteins in PaTu8988 cells with transfected with indicated constructs (D) and quantification (C; n = 3 biologically independent samples) of gray value of HA-HSF1/Flag-HSF1. **(E)** Quantification of gray value of AcK/ HSF1 in Figure 5A. **(F)** Quantification of gray value of AcK/ HSF1 in Figure 5B. **(G)** Quantification of gray value of SIRT1/ HSF1 in Figure 5C. **(H)** Quantification of gray value of BAP1/ HSF1 in Figure 5D. **(I)** Quantification of gray value of BAP1/ Flag-HSF1 in Figure 5E.

**Supplementary Table S1. Information of recombinant DNA**

| **Recombinant DNA** | **Source** | **Identifier** |
| --- | --- | --- |
| Flag-HSF1 | Addgene | 32537 |
| SFB-BAP1/SFB-BAP1(C91A)/SFB-BAP1(C91S)/SFB-EV were a gift of Huang’s lab (Mayo Clinic). | | |

**Supplementary Table S2. Sequence of primers and gene specific shRNAs & SiRNAs**

| **Gene** | **Usage** | **Forward** | **Reverse** |
| --- | --- | --- | --- |
| *GAPDH* | RT-qPCR | GTCAACGGATTTGGTCGTAT | GAACATGTAAACCATGTAGTTGA |
| *Gapdh* | RT-qPCR | CGACTTCAACAGCAACTCCCACTCTTCC | TGGGTGGTCCAGGGTTTCTTACTCCTT |
| *BAP1* | RT-qPCR | GACCCAGGCCTCTTCACC | AGTCCTTCATGCGACTCAGG |
| *Bap1* | RT-qPCR | GGATTGAAAGTCTACCCAATTGAT | CGAGCTTTATCTGTCCACTCCT |
| *HYPK* | RT-qPCR | GAATGCGGCGGCGTGGTG | TCAGTTGGTTAGGGCAATAAGC |
| *HSF1* | RT-qPCR | GACCAAGCTGTGGACCCTC | CACTTTCCGGAAGCCATACAT |
| *STIP1* | RT-qPCR | GCCAAGCGAACCTATGAGGAG | GGATCACTGAGTAGTGTCCTTGT |
| *JUN* | RT-qPCR | GAGCTGGAGCGCCTGATAAT | CCCTCCTGCTCATCTGTCAC |
| *CREB5* | RT-qPCR | ATCACCAGACCTCGCCACAT | GCTGGGGTGGCTGTATTGTC |
| *ATF4* | RT-qPCR | CTCTTGACCACGTTGGATGAC | CAACTTCACTGCCTAGCTCTAAA |
| *STIP1 promoter* | ChIP-qPCR | GGCTACGATTGGCAGTGCA | GGAGCGAACTTCTGCGACAC |
| *HYPK promoter* | ChIP-qPCR | ATCCCAAATGAGAGGGGGTTG | TTGCTGTGTAACCTTGAGCCA |
| *ATF4*  *promoter* | ChIP-qPCR | TGGGCATAAACGGTTGGGG | CTATGGGGACGCAGCACAGA |
| *CREB5*  *promoter* | ChIP-qPCR | CCCAGTGTGTGTTGTTCCTCT | GGCTATTGCAGCTAATTCTCGG |
| **shRNAs** | **Sequence** | | |
| sh-SIRT1-1 | CTAGGAATGTTGAAAGTATTG | | |
| sh-SIRT1-2 | CCATGAAGTATGACAAAGATG | | |
| sh-HSF1-1 | GCAGGTTGTTCATAGTCAGAAC | | |
| sh-HSF1-2 | GCCCAAGTACTTCAAGCACAAC | | |
| shBAP1-1/2 were a gift of Huang’s lab (Mayo Clinic). | | | |

**Supplementary Table S3. Information of antibodies**

| **Antibodies** | **Source** | **Identifier** | **Working dilution** |
| --- | --- | --- | --- |
| Rabbit polyclonal anti-HA-tag Antibody | Proteintech | Cat # 51064-2-AP;  RRID: AB_11042321 | 1:1000 |
| Rabbit polyclonal anti-GAPDH Antibody | Proteintech | Cat #10494-1-AP; RRID: AB_2263076 | 1:3000 |
| Rabbit polyclonal anti-BAP1 Antibody | Proteintech | Cat # 10398-1-AP; RRID: AB_2180460 | 1:1000 |
| PD-1/CD279 Monoclonal antibody | Proteintech | Cat # 66220-1-Ig; RRID: AB_2881611 | 1:10000 |
| PD-L1/CD274 Polyclonal antibody | Proteintech | Cat # 17952-1-AP ; RRID: AB_10597552 | 1:1000 |
| Rabbit Polyclonal anti-Flag-tag Antibody | Proteintech | Cat # 20543-1-AP; RRID: AB_11232216 | 1:1000 |
| Rabbit Polyclonal anti- HSF1 Antibody | Cell Signaling Technology | Cat# 4356;  RRID: AB_2120258 | 1:1000 |
| Rabbit Polyclonal anti-MYC-tag Antibody | Proteintech | Cat# 16286-1-AP; RRID: AB_11182162 | 1:1000 |
| Rabbit Polyclonal anti-SIRT1 Antibody | Proteintech | Cat#13161-1-AP; RRID: AB_10646436 | 1:1000 |
| Acetylated-Lysine Antibody | Cell Signaling Technology | Cat#9441; RRID: AB_331805 | 1:1000 |
| HRP Conjugated AffiniPure Goat Anti-Mouse IgG (H+L) | Boster Biological Technology | Cat#BA1050;  RRID: AB_2904507 | 1:4000 |
| Mouse anti-rabbit IgG (Conformation specific) monoclonal antibody (HRP conjugate) | Cell Signaling Technology | Cat# 5127;  RRID: AB_10892860 | 1:4000 |
| APC anti-mouse CD45 Antibody | Biolegend | Cat# 103112;  RRID: AB_312976 | 0.2 µg per 10^6^ cells in 100 µl |
| FITC anti-mouse CD4 Antibody | Biolegend | Cat# 100510;  RRID: AB_312713 | 0.2 µg per 10^6^ cells in 100 µl |
| PE anti-mouse CD8a Antibody | Biolegend | Cat#100708;  RRID: AB_312747 | 0.2 µg per 10^6^ cells in 100 µl |
| APC anti-mouse/human CD11b Antibody | Biolegend | Cat#101212;  RRID: AB_312795 | 0.2 µg per 10^6^ cells in 100 µl |
| FITC anti-mouse Ly-6G/Ly-6C (Gr-1) Antibody | Biolegend | Cat#108406;  RRID: AB_313370 | 0.2 µg per 10^6^ cells in 100 µl |

**Supplementary Table S4. Information of chemicals and kits**

| **Chemicals** | **Source** | **Identifier** |
| --- | --- | --- |
| DMSO | Sorlabio | [D8371](https://www.solarbio.com/goods-370.html) |
| Lipo8000™ Transfection Reagent | Beyotime | C0533 |
| Opti-MEM | Gibco | 11058021 |
| Polybrene | Beyotime | C0351-1ml |
| [Puromycin Dihydrochloride](https://www.beyotime.com/product/ST551-10mg.htm) | Beyotime | ST551 |
| DMEM | Gibco | 11965092 |
| PBS | Gibco | 20012050 |
| Fetal Bovein Serum（FBS） | Gibco | 10099141 |
| RIPA lysis buffer | Beyotime | P0013B |
| TRIzol reagent | Thermo Fisher Scientific | 15596018 |
| PMSF protease inhibitor | Beyotime | P1046 |
| Protein A/G agarose beads | Beyotime | P2055 |
| Phosphatase inhibitor A | Beyotime | P1082 |
| Phosphatase inhibitor B | Beyotime | P1087 |
| Lookout Mycoplasma PCR Detection Kit | Sigma-Aldrich | MP0035 |
| PrimeScript™ RT Reagent Kit | Takara Bio Inc. | RR037A |
| TB Green™ Fast qPCR Mix PCR Kit | Takara Bio Inc. | RR430A |
| BCA Protein Assay Kit | Beyotime | P0012S |
| Tween-20 | Biosharp | BS100 |
| TBS | Servicebio | G0001 |
| Triton X-100 | Biosharp | BS084 |
| EX-527 | MedChemExpress | HY-15452 |
| HSF1 Luciferase Reporter Lentivirus | Creative Biogene | LVG00095Z |
