# Supplementary material for: BAP1 regulates HSF1 activity and cancer immunity in pancreatic cancer
Source: J Exp Clin Cancer Res. 2024 Sep 30;43:275. doi: 10.1186/s13046-024-03196-4 (PMC11441124; doi:10.1186/s13046-024-03196-4)
Supplement: Supplementary file 5 — Supplementary Material 5 [file 13046_2024_3196_MOESM5_ESM.docx]

**Supplementary Table S1. Information of recombinant DNA**

| **Recombinant DNA** | **Source** | **Identifier** |
| --- | --- | --- |
| Flag-HSF1 | Addgene | 32537 |
| SFB-BAP1/SFB-BAP1(C91A)/SFB-BAP1(C91S)/SFB-EV were a gift of Huang’s lab (Mayo Clinic). | | |
