# Supplementary material for: BAP1 regulates HSF1 activity and cancer immunity in pancreatic cancer
Source: J Exp Clin Cancer Res. 2024 Sep 30;43:275. doi: 10.1186/s13046-024-03196-4 (PMC11441124; doi:10.1186/s13046-024-03196-4)
Supplement: Supplementary file 6 — Supplementary Material 6 [file 13046_2024_3196_MOESM6_ESM.docx]

**Supplementary Table S2. Sequence of primers and gene specific shRNAs & SiRNAs**

| **Gene** | **Usage** | **Forward** | **Reverse** |
| --- | --- | --- | --- |
| *GAPDH* | RT-qPCR | GTCAACGGATTTGGTCGTAT | GAACATGTAAACCATGTAGTTGA |
| *Gapdh* | RT-qPCR | CGACTTCAACAGCAACTCCCACTCTTCC | TGGGTGGTCCAGGGTTTCTTACTCCTT |
| *BAP1* | RT-qPCR | GACCCAGGCCTCTTCACC | AGTCCTTCATGCGACTCAGG |
| *Bap1* | RT-qPCR | GGATTGAAAGTCTACCCAATTGAT | CGAGCTTTATCTGTCCACTCCT |
| *HYPK* | RT-qPCR | GAATGCGGCGGCGTGGTG | TCAGTTGGTTAGGGCAATAAGC |
| *HSF1* | RT-qPCR | GACCAAGCTGTGGACCCTC | CACTTTCCGGAAGCCATACAT |
| *STIP1* | RT-qPCR | GCCAAGCGAACCTATGAGGAG | GGATCACTGAGTAGTGTCCTTGT |
| *JUN* | RT-qPCR | GAGCTGGAGCGCCTGATAAT | CCCTCCTGCTCATCTGTCAC |
| *CREB5* | RT-qPCR | ATCACCAGACCTCGCCACAT | GCTGGGGTGGCTGTATTGTC |
| *ATF4* | RT-qPCR | CTCTTGACCACGTTGGATGAC | CAACTTCACTGCCTAGCTCTAAA |
| *STIP1 promoter* | ChIP-qPCR | GGCTACGATTGGCAGTGCA | GGAGCGAACTTCTGCGACAC |
| *HYPK promoter* | ChIP-qPCR | ATCCCAAATGAGAGGGGGTTG | TTGCTGTGTAACCTTGAGCCA |
| *ATF4*  *promoter* | ChIP-qPCR | TGGGCATAAACGGTTGGGG | CTATGGGGACGCAGCACAGA |
| *CREB5*  *promoter* | ChIP-qPCR | CCCAGTGTGTGTTGTTCCTCT | GGCTATTGCAGCTAATTCTCGG |
| **shRNAs** | **Sequence** | | |
| sh-SIRT1-1 | CTAGGAATGTTGAAAGTATTG | | |
| sh-SIRT1-2 | CCATGAAGTATGACAAAGATG | | |
| sh-HSF1-1 | GCAGGTTGTTCATAGTCAGAAC | | |
| sh-HSF1-2 | GCCCAAGTACTTCAAGCACAAC | | |
| shBAP1-1/2 were a gift of Huang’s lab (Mayo Clinic). | | | |
