# Supplementary material for: BAP1 regulates HSF1 activity and cancer immunity in pancreatic cancer
Source: J Exp Clin Cancer Res. 2024 Sep 30;43:275. doi: 10.1186/s13046-024-03196-4 (PMC11441124; doi:10.1186/s13046-024-03196-4)
Supplement: Supplementary file 7 — Supplementary Material 7 [file 13046_2024_3196_MOESM7_ESM.docx]

**Supplementary Table S3. Information of antibodies**

| **Antibodies** | **Source** | **Identifier** | **Working dilution** |
| --- | --- | --- | --- |
| Rabbit polyclonal anti-HA-tag Antibody | Proteintech | Cat # 51064-2-AP;  RRID: AB_11042321 | 1:1000 |
| Rabbit polyclonal anti-GAPDH Antibody | Proteintech | Cat #10494-1-AP; RRID: AB_2263076 | 1:3000 |
| Rabbit polyclonal anti-BAP1 Antibody | Proteintech | Cat # 10398-1-AP; RRID: AB_2180460 | 1:1000 |
| PD-1/CD279 Monoclonal antibody | Proteintech | Cat # 66220-1-Ig; RRID: AB_2881611 | 1:10000 |
| PD-L1/CD274 Polyclonal antibody | Proteintech | Cat # 17952-1-AP ; RRID: AB_10597552 | 1:1000 |
| Rabbit Polyclonal anti-Flag-tag Antibody | Proteintech | Cat # 20543-1-AP; RRID: AB_11232216 | 1:1000 |
| Rabbit Polyclonal anti- HSF1 Antibody | Cell Signaling Technology | Cat# 4356;  RRID: AB_2120258 | 1:1000 |
| Rabbit Polyclonal anti-MYC-tag Antibody | Proteintech | Cat# 16286-1-AP; RRID: AB_11182162 | 1:1000 |
| Rabbit Polyclonal anti-SIRT1 Antibody | Proteintech | Cat#13161-1-AP; RRID: AB_10646436 | 1:1000 |
| Acetylated-Lysine Antibody | Cell Signaling Technology | Cat#9441; RRID: AB_331805 | 1:1000 |
| HRP Conjugated AffiniPure Goat Anti-Mouse IgG (H+L) | Boster Biological Technology | Cat#BA1050;  RRID: AB_2904507 | 1:4000 |
| Mouse anti-rabbit IgG (Conformation specific) monoclonal antibody (HRP conjugate) | Cell Signaling Technology | Cat# 5127;  RRID: AB_10892860 | 1:4000 |
| APC anti-mouse CD45 Antibody | Biolegend | Cat# 103112;  RRID: AB_312976 | 0.2 µg per 10^6^ cells in 100 µl |
| FITC anti-mouse CD4 Antibody | Biolegend | Cat# 100510;  RRID: AB_312713 | 0.2 µg per 10^6^ cells in 100 µl |
| PE anti-mouse CD8a Antibody | Biolegend | Cat#100708;  RRID: AB_312747 | 0.2 µg per 10^6^ cells in 100 µl |
| APC anti-mouse/human CD11b Antibody | Biolegend | Cat#101212;  RRID: AB_312795 | 0.2 µg per 10^6^ cells in 100 µl |
| FITC anti-mouse Ly-6G/Ly-6C (Gr-1) Antibody | Biolegend | Cat#108406;  RRID: AB_313370 | 0.2 µg per 10^6^ cells in 100 µl |
