# Supplementary material for: BAP1 regulates HSF1 activity and cancer immunity in pancreatic cancer
Source: J Exp Clin Cancer Res. 2024 Sep 30;43:275. doi: 10.1186/s13046-024-03196-4 (PMC11441124; doi:10.1186/s13046-024-03196-4)
Supplement: Supplementary file 8 — Supplementary Material 8 [file 13046_2024_3196_MOESM8_ESM.docx]

**Supplementary Table S4. Information of chemicals and kits**

| **Chemicals** | **Source** | **Identifier** |
| --- | --- | --- |
| DMSO | Sorlabio | [D8371](https://www.solarbio.com/goods-370.html) |
| Lipo8000™ Transfection Reagent | Beyotime | C0533 |
| Opti-MEM | Gibco | 11058021 |
| Polybrene | Beyotime | C0351-1ml |
| [Puromycin Dihydrochloride](https://www.beyotime.com/product/ST551-10mg.htm) | Beyotime | ST551 |
| DMEM | Gibco | 11965092 |
| PBS | Gibco | 20012050 |
| Fetal Bovein Serum（FBS） | Gibco | 10099141 |
| RIPA lysis buffer | Beyotime | P0013B |
| TRIzol reagent | Thermo Fisher Scientific | 15596018 |
| PMSF protease inhibitor | Beyotime | P1046 |
| Protein A/G agarose beads | Beyotime | P2055 |
| Phosphatase inhibitor A | Beyotime | P1082 |
| Phosphatase inhibitor B | Beyotime | P1087 |
| Lookout Mycoplasma PCR Detection Kit | Sigma-Aldrich | MP0035 |
| PrimeScript™ RT Reagent Kit | Takara Bio Inc. | RR037A |
| TB Green™ Fast qPCR Mix PCR Kit | Takara Bio Inc. | RR430A |
| BCA Protein Assay Kit | Beyotime | P0012S |
| Tween-20 | Biosharp | BS100 |
| TBS | Servicebio | G0001 |
| Triton X-100 | Biosharp | BS084 |
| EX-527 | MedChemExpress | HY-15452 |
| HSF1 Luciferase Reporter Lentivirus | Creative Biogene | LVG00095Z |
